# Synthesis and applications of alkenyl chlorides (vinyl chlorides): a review

**DOI:** 10.3762/bjoc.22.1

**Published:** 2026-01-02

**Authors:** Daniel S Müller

**Affiliations:** 1 Univ. Rennes, CNRS, ISCR (Institut des Sciences Chimiques de Rennes) – UMR 6226, 263 Avenue du Général Leclerc, F-35000 Rennes, France

**Keywords:** alkenyl chloride, chloroalkenes, chloro olefins, vinyl chlorides

## Abstract

Alkenyl chlorides constitute a synthetically valuable yet historically underexplored class of organohalides. First prepared in 1868 by Charles Friedel – best known for the Friedel–Crafts reaction – via the reaction of ketones with phosphorus pentachloride, these compounds have steadily gained attention over the decades. In recent years, their distinct reactivity and potential in organic synthesis have been increasingly recognized. This review provides a comprehensive overview of the synthesis and application of alkenyl chlorides, with a focus on developments over the past four decades. By organizing this growing body of work, I aim to highlight key advances and help guide the design of new transformations involving this important and versatile functional group.

## Introduction

**Figure 1:**
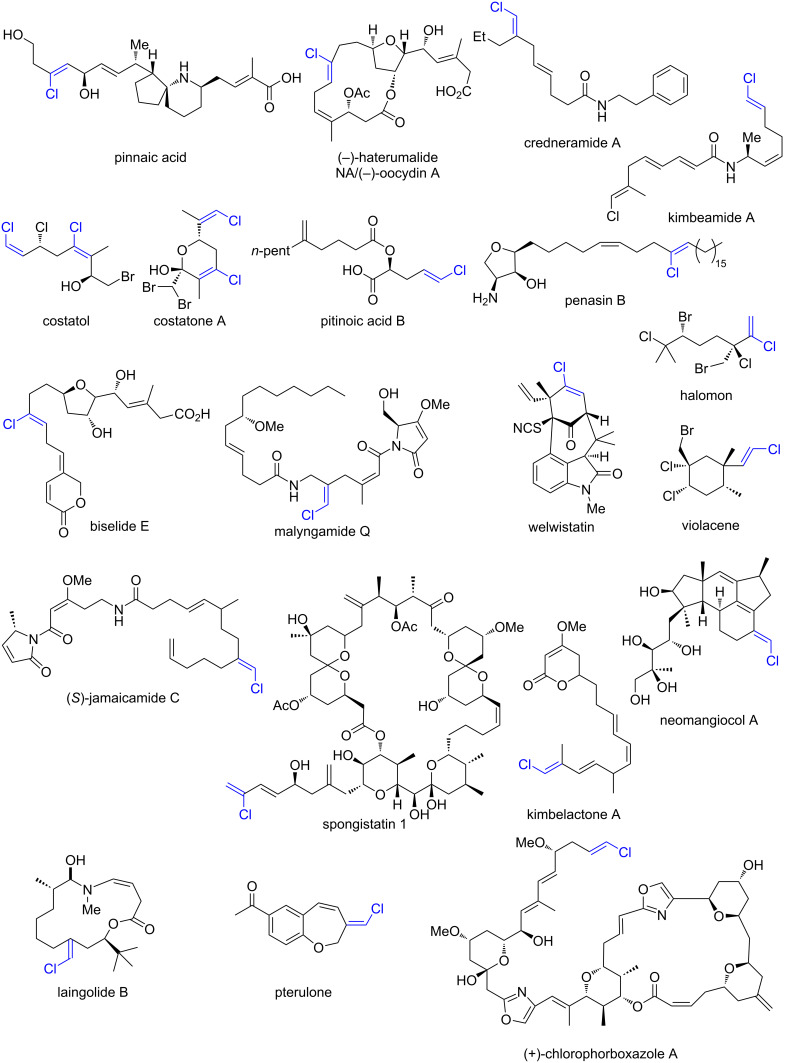
Representative alkenyl chloride motifs in natural products. References: Pinnaic acid [8], haterumalide [9], credneramide A [10], kimbeamide A [11], costatol [12], costatone A [12], pitinoic acid B [13], penasin B [14], halomon [15], biselide E [16], malyngamide Q [17], welwistatin [18], violacene [19], jamaicamide C [20], spongistatin 1 [21], kimbelactone A [11], neomangiocol A [22], laingolide B [23], pterulone [24], (+)-chlorophorboxazole A [21].

**Figure 2:**
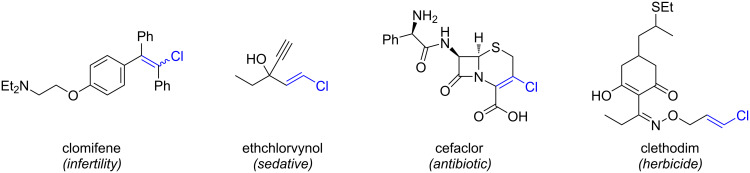
Representative alkenyl chloride motifs in pharmaceuticals and pesticides. References: clomifene [25], ethchlorvynol [26], cefaclor [27], clethodim [28].

Alkenyl chlorides, while less extensively investigated than their brominated analogues, constitute a synthetically valuable class of organohalides with distinct reactivity. Among halogens, chloride is unique in its virtually limitless availability, derived from the global abundance of sodium chloride. In contrast, bromide and iodide sources are geographically restricted – bromide is predominantly extracted from the Dead Sea [1], while iodide is primarily sourced from caliche deposits in Chile [2]. This concentration of supply raises potential concerns regarding long-term availability and geopolitical vulnerability. Recent interest in alkenyl chlorides has been driven by their occurrence in bioactive natural products (Figure 1) [3–5], pharmaceuticals (Figure 2) [6], and pesticides [7] (Figure 2). Throughout this review, the term “alkenyl chloride” refers broadly to chloroalkenes beyond vinyl chloride (CH_2_=CHCl), the monomer used in polyvinyl chloride (PVC) production.

**Figure 3:**
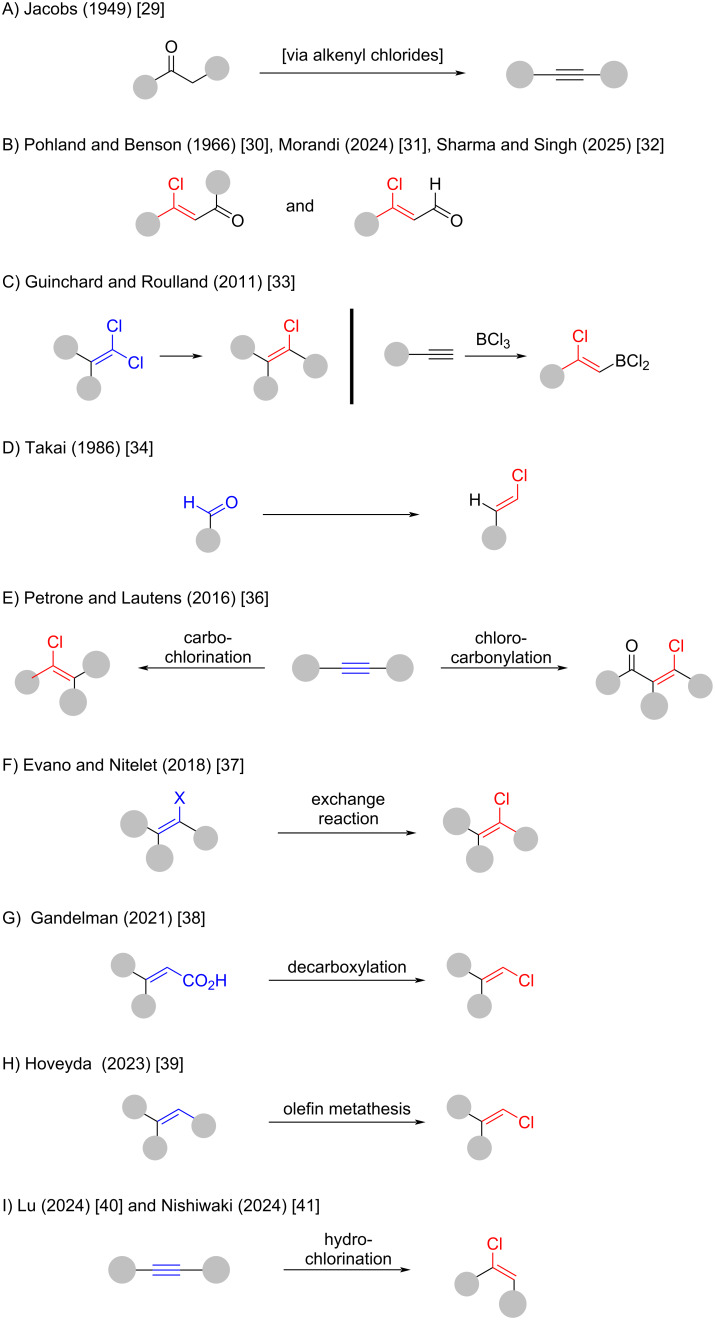
Graphical overview of previously published reviews addressing the synthesis of alkenyl chlorides.

Before detailing the synthesis and applications of alkenyl chlorides, we wish to acknowledge previous reviews that have addressed this class of compounds in part or within broader contexts. Figure 3 offers a structured overview of prior reviews to facilitate orientation within the existing literature. The earliest comprehensive account is found in Jacobs’ 1949 Organic Reactions chapter, “The Synthesis of Acetylenes” [29] which discusses the preparation of alkenyl chlorides via ketone chlorination using PCl_5_ (Figure 3A). The synthesis of β-chlorovinyl ketones was later reviewed by Poland and Benson in 1966 (Figure 3B) [30]. More recently, Morandi and co-workers summarized transition-metal-catalyzed additions of alkynes to acyl chlorides to access β-chlorovinyl ketones (2024) [31]. A review by Sharma and Singh covers comprehensively the synthesis and application of β-halovinyl aldehydes [32]. It should be noted that the synthesis of β-chloroalkenes substituted with an electron-withdrawing group in the α-position (e.g., aldehyde, ketone, sulfone, nitro) is beyond the scope of this review. In 2011, Guinchard and Roulland reviewed Pd-catalyzed cross-couplings of 1,1-dichloroalkenes and boron-chlorination reactions in the context of natural product synthesis (Figure 3C) [33]. Takai’s contribution to the field, particularly via chromium-mediated olefinations, is covered in a Comprehensive Organic Synthesis chapter (Figure 3D) [34–35]. Carbochlorination and carbonylchlorination reactions were reviewed by Petrone, Ye, and Lautens in their Chemical Reviews article on transition-metal-catalyzed C–halogen bond formation (Figure 3E) [36]. Exchange reactions were reviewed by Evano and Nitelet in 2018 (Figure 3F) [37]. In 2020, Gandelman and co-workers provided an overview of decarboxylative chlorination reactions of carboxylic acids (Figure 3G) [38]. Hoveyda’s 2023 review highlights stereocontrolled olefin metathesis with molybdenum catalysts to access trisubstituted alkenes including alkenyl chlorides (Figure 3H) [39]. Two recent reviews by Lu [40] and Nishiwaki [41] provide overviews of current developments in the hydrochlorination of alkynes, highlighting emerging catalytic strategies (Figure 3I). It should be noted that the review by Petrone and Lautens also covers some hydrochlorination chemistry. A concise three-page overview of alkenyl chloride synthesis appeared in 1995 in a book chapter “*Comprehensive Organic Functional Group Transformations”* by C. J. Urch [42]. Lastly, Cao and co-workers published a mini-review in 2013, written in Chinese, summarizing advances in the synthesis of vinyl chlorides [43].

**Figure 4:**
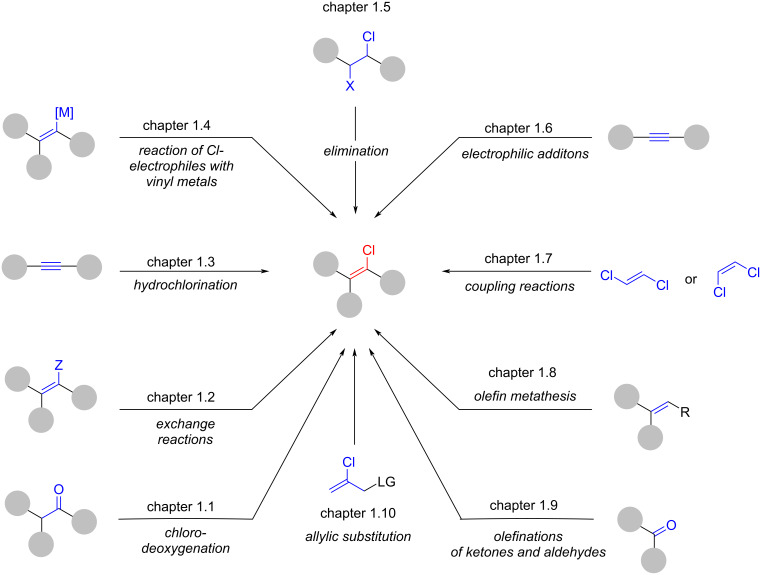
Classification of synthetic approaches to alkenyl chlorides.

As illustrated by the summary of existing reviews (Figure 3), the synthesis of alkenyl chlorides encompasses a wide range of mechanistically distinct strategies. An overview and classification of these transformations – excluding decarboxylative processes – is provided in Figure 4. Each class is discussed in detail in chapter 1. However, several transformations do not fit cleanly into the defined categories and are therefore discussed under miscellaneous reactions (chapter 1.11). For areas already covered by prior reviews, repetition has been avoided unless inclusion of key historical reports, overlooked publications, or representative examples was deemed necessary to highlight fundamental concepts.

Given the extensive literature on the synthesis of alkenyl halides – including iodides, bromides, and chlorides – this review is limited to studies that report at least two distinct examples of alkenyl chloride formation. The preparation of 1,1-dichloroalkenes has been comprehensively reviewed by Chelucci and will not be revisited here [44]. Conversely, 1,2-dichloroalkene synthesis lies beyond the scope of the present discussion. Regarding the use of color in graphical representations, structural highlighting was applied selectively, only when judged necessary in the clear identification of the transformation or mechanism depicted.

Chapter 2 highlights key applications of alkenyl chlorides. The discussion begins with reductive metalation, reactions with organolithium reagents, and eliminations to terminal alkynes – some of which are also exemplified in chapter 1. This is followed by palladium- and nickel-catalyzed cross-coupling reactions. The chapter concludes with a selection of miscellaneous transformations illustrating the broader synthetic utility of this class of compounds.

This review aims to provide a comprehensive overview of synthetic approaches to alkenyl chlorides and applications thereof. However, as the literature proved vast and ever-expanding, some reports may have escaped our attention and certain compound classes remain beyond the scope of this article. Still, with over 200 references, we hope to offer a representative and valuable perspective on the field.

## Review

### 1 Synthesis of alkenyl chlorides

#### 1.1 Chlorodeoxygenations

**Scheme 1:**
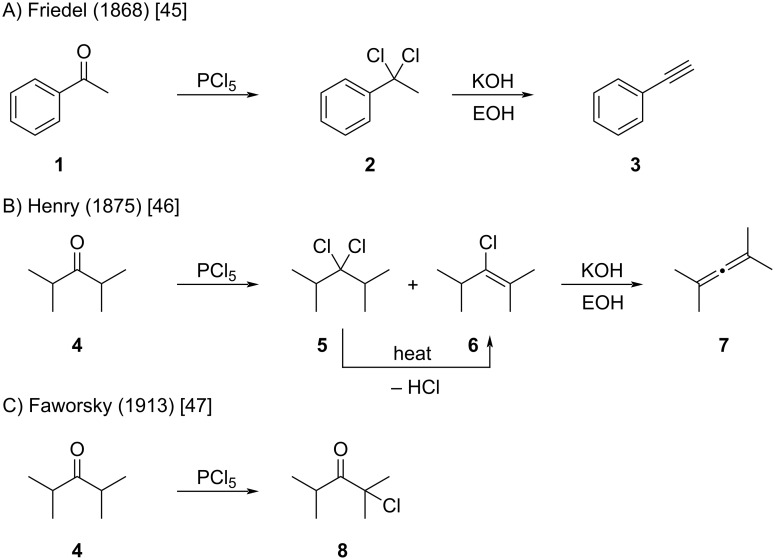
Early works by Friedel, Henry, and Favorsky.

**Transformations of ketones to alkenyl chlorides with phosphorous pentachloride (PCl_5_):** Friedel first reported the reaction of PCl_5_ with acetophenone (**1**) in 1868 (Scheme 1A) [45]. Treatment of the resulting intermediate **2 –** then presumed to be a *gem*-dichloride – with aqueous KOH led to the formation of phenylacetylene (**3**). In 1875, Louis Henry extended this transformation to ketone **4** (Scheme 1B) [46]. Exposure to PCl_5_ gave a mixture of chlorinated intermediates described as **5** and **6**, which, upon prolonged treatment with ethanolic KOH, underwent elimination to afford allene **7**. Henry also observed that thermal treatment of the dichloro intermediate **5** yielded the corresponding alkenyl chloride **6**. In 1913, Faworsky revisited this transformation in an effort to prepare tetramethylallene (**7**) (Scheme 1C) [47]. However, several attempts to reproduce Henry’s procedure were unsuccessful. Instead, Faworsky isolated the α-chlorinated ketone **8** as the major product. To our knowledge, the exact cause of this divergent chemoselectivity observed by Henry and Faworsky has never been discussed in the literature.

**Scheme 2:**
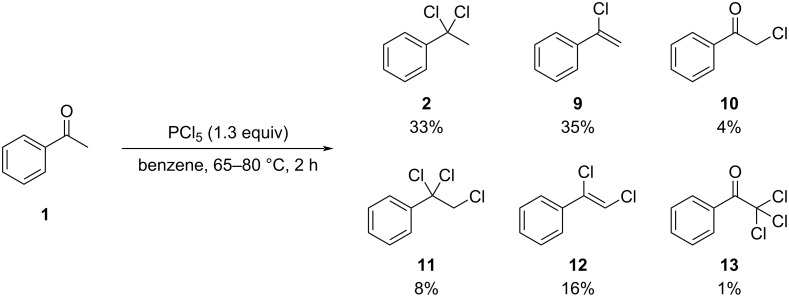
Product distribution obtained by H NMR integration of crude compound as observed by Kagan and co-workers.

Numerous reports describe the formation of alkenyl chlorides via treatment of ketones with PCl_5_. Notably, the reaction conditions vary widely across the literature, with solvents ranging from neat conditions to cyclohexane, hexanes, toluene, benzene, carbon tetrachloride, dichloromethane, and diethyl ether. Reaction temperatures span from −10 °C to 100 °C. The first detailed investigation of this transformation was reported by Kagan and co-workers [48], who demonstrated that the reaction of acetophenone (**1**) with PCl_5_ in refluxing benzene affords a complex product mixture (Scheme 2).

**Scheme 3:**
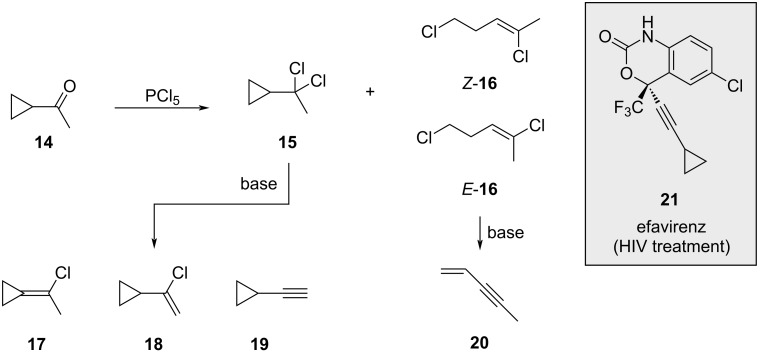
Side reactions observed for the reaction of **14** with PCl_5_.

A second, very detailed investigation of this reaction was presented by Jung and Kwon [49]. Focusing on the synthesis of efavirenz (**21**), a potent HIV-1 non-nucleoside reverse transcriptase inhibitor, they aimed to improve the preparation of compound **19**, whose yield had previously been limited to 22% following a similar route. They noticed that in situ-generated species such as POCl_3_ or HCl triggered ring opening of dichloride **15** to produce a mixture of alkenyl chlorides *Z*- and *E*-**16** (Scheme 3).

**Scheme 4:**
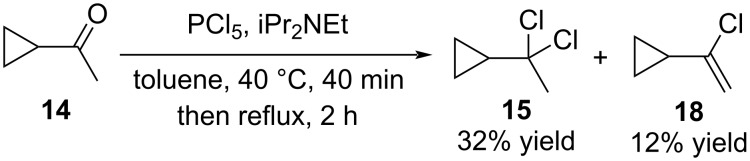
Only compounds **15** and **18** were observed in the presence of Hünig’s base.

Treatment of the reaction mixture with base resulted in the formation of four distinct products (**17**, **18**, **19** and **20**) that proved challenging to separate. Screening of various bases revealed Hünig’s base (iPr_2_NEt) as uniquely effective in producing compounds **15** and **18** without contamination with *E*- or *Z*-**16** isomers (Scheme 4). Additionally, it was found that several aqueous work-up procedures induced ring opening of compounds **17** and **18**. Ultimately, steam distillation directly from the reaction mixture afforded a toluene solution of compounds **15** and **18** in a combined yield of 44% (Scheme 4).

**Scheme 5:**
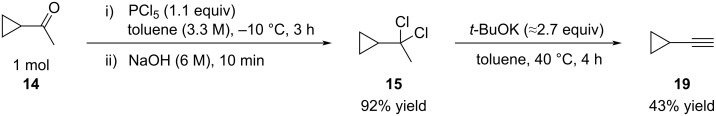
Efficient synthesis of dichloride **15** at low temperatures.

Additional optimization reactions showed that when the reaction was carried out at −10 °C even in the absence of Hünig’s base dichloride **15** could be obtained in 92% yield. Treatment of **15** with KO*t-*Bu in toluene gave the desired compound **19** in 43% yield. The authors justified the low yield by the high volatility of **19** and loss thereof during work-up (Scheme 5).

It should be noted that the two-step procedure for converting a ketone into the corresponding alkyne can be accomplished in a single step, as recently reported by Ghaffarzadeh and co-workers) [50]. They demonstrated that a 9:1 molar ratio of pyridine to PCl_5_ converts ketones to alkynes within minutes under microwave irradiation (not shown).

**Scheme 6:**
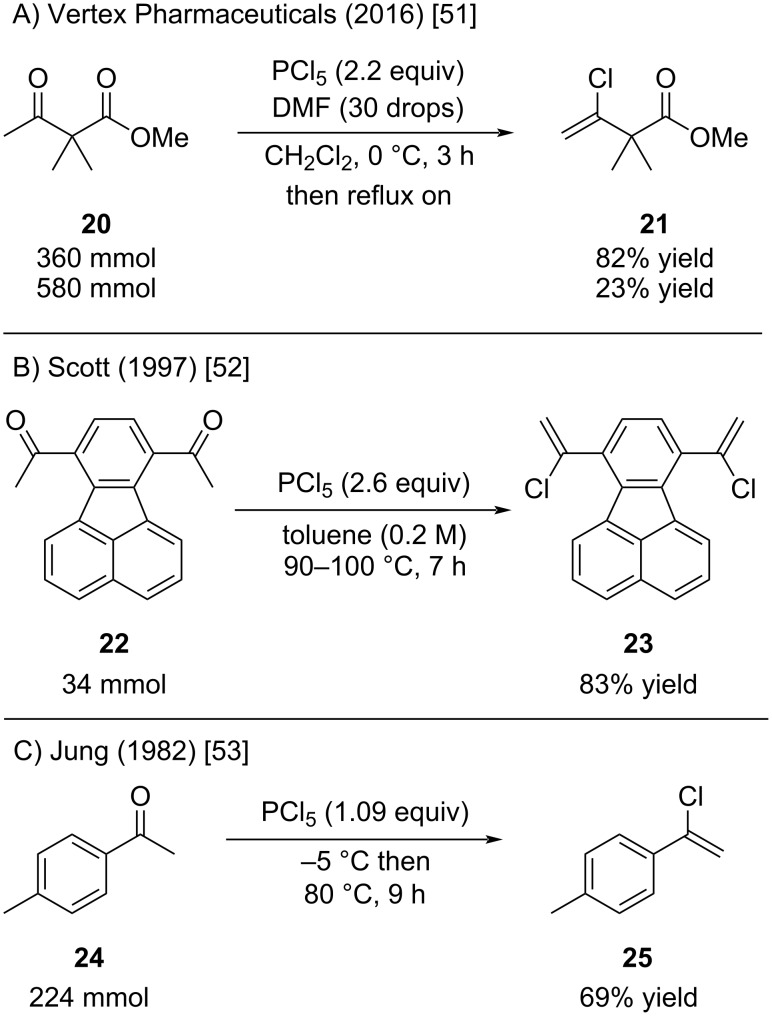
Various syntheses of alkenyl chlorides on larger scale.

High yields for alkenyl chlorides from ketones by reaction with PCl_5_ were reported under various conditions on preparative scales. For instance, a patent from Vertex Pharmaceuticals describes the synthesis of alkenyl chloride **21** on a 360 and 580 mmol scale (Scheme 6A) [51]. The addition of a few drops of DMF was not commented. Surprisingly a significant decrease in yield was observed at the larger 580 mmol scale compared to the 360 mmol scale. Scott reported a high-yielding synthesis of **23** by heating diketone **22** with PCl_5_ for several hours in toluene (Scheme 6B) [52]. Similarly, Jung obtained **25** in 69% yield after heating neat ketone and PCl_5_ (Scheme 6C) [53]. The high yield of **25** is somewhat surprising, considering the limited stability thereof. In our laboratory, **25** decomposed completely to a black tar within days, even when stored at −20 °C.

**Scheme 7:**
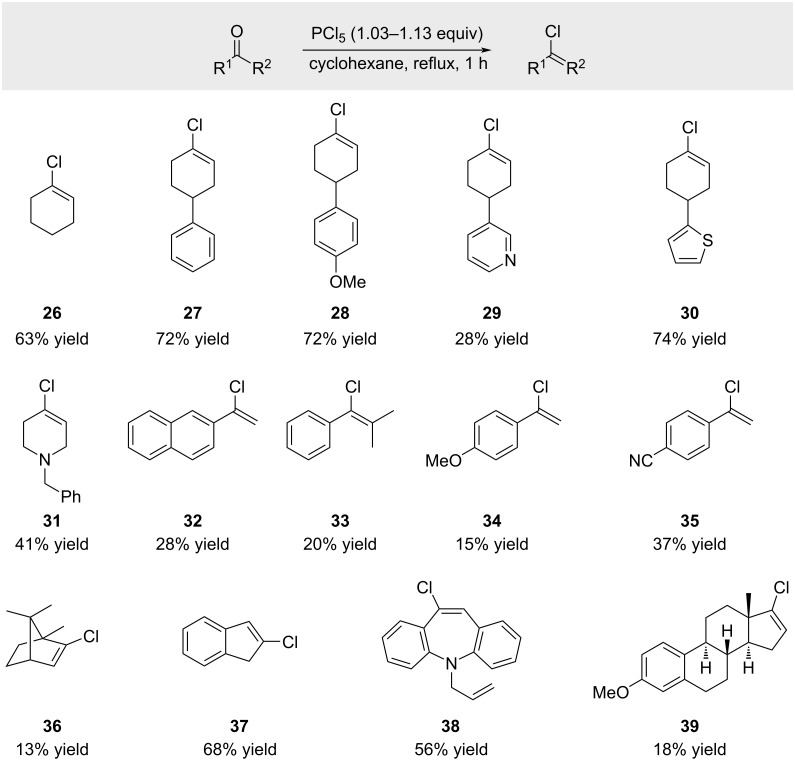
Scope of the reaction of ketones with PCl_5_ in boiling cyclohexane.

Several research groups reported the transformation of a broad range of ketones into the corresponding alkenyl chlorides with PCl_5_ in boiling cyclohexanes (Scheme 7; product **26** [54], products **27**, **36**, and **37** [55] and products **28**–**35**, **38** and **39** [56]). The yields varied considerably depending on the substrate, leading to the following conclusions: (a) unhindered ketones lacking functional groups generally react in high yields; (b) sterically hindered ketones provide products **33**, **36**, and **39** with low yields; (c) electron-rich acetophenone derivatives, such as the *p*-methoxy-substituted example **34** afforded only low yields.

**Scheme 8:**
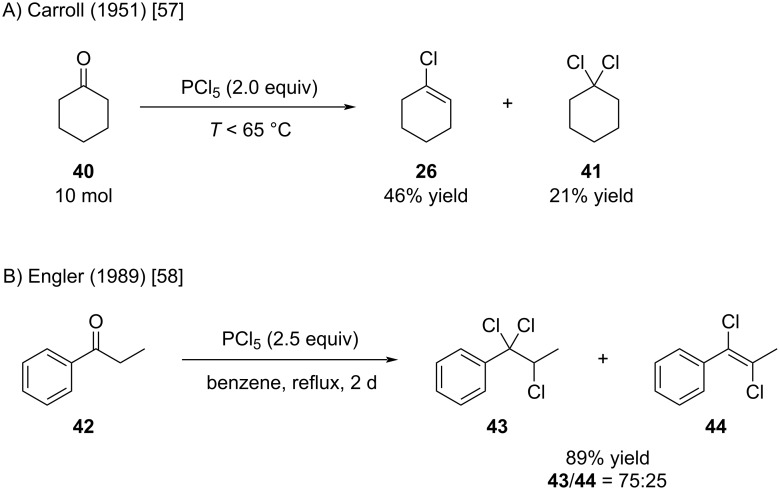
Side reactions occur when using excess amounts of PCl_5_.

It is noteworthy that performing the synthesis of **26** under neat conditions with an excess of PCl_5_ afforded substantial amounts of dichloride **41** (Scheme 8A) [57]. Engler showed that the use of a large excess of PCl_5_ and prolonged reaction times can even favor the formation of trichloride **43** (Scheme 8B) [58].

Finally, it should be noted that, in contrast to five- and six-membered ring systems, Nagendrappa reported that medium-ring cycloalkanones afforded complex product mixtures, with the desired alkenyl chlorides isolated in purities below 85% (not shown) [59].

**Scheme 9:**
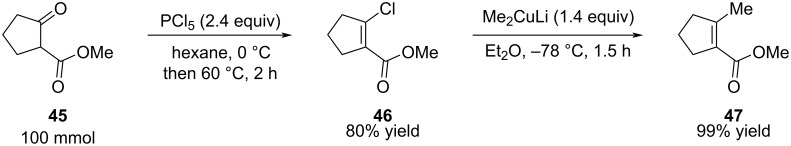
Formation of versatile β-chlorovinyl ketones.

**Transformations of enols to alkenyl chlorides with phosphorous pentachloride (PCl_5_):** β-Chlorovinyl ketones and esters represent highly versatile intermediates, as the chloride moiety is readily displaced by a wide range of nucleophiles (for example Scheme 9) [60]. These compounds are efficiently obtained in high yields from the corresponding ketones by treatment with PCl_5_. Interestingly, an excess of PCl_5_ does not lead to significant amounts of undesired side products. For excellent reviews concerning the synthesis of β-chlorovinyl ketones the reader is referred to Figure 3B and references mentioned therein.

**Scheme 10:**
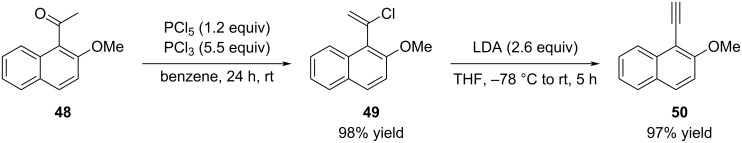
Mixture of PCl_5_ and PCl_3_ used for the synthesis of **49**.

**Other phosphorus-based procedures:** Heller reported the transformation of **48** to **49** using a mixture of PCl_5_ and PCl_3_ [61]. The authors did not comment on PCl_3_ as additive. Both, the formation of the alkenyl chloride and the corresponding alkyne **50** by elimination with LDA were achieved in superb yields (Scheme 10).

**Scheme 11:**
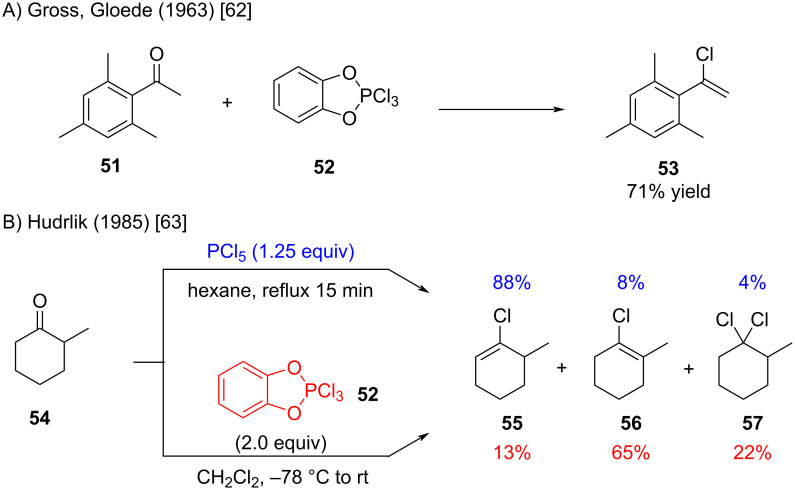
Catechol–PCl_3_ reagents for the synthesis of alkenyl chlorides.

Gross and Gloede introduced catechol–PCl_3_ as an effective reagent for the conversion of ketones to the corresponding alkenyl chlorides [62] (Scheme 11A). Hudrlik later demonstrated that, in the case of 2-methylcyclohexanone (**54**), this reagent markedly alters the product distribution compared to PCl_5_ [63] (Scheme 11B). However, since the reaction conditions differ significantly, it remains unclear whether the product distribution is driven by the reagent itself or by the reaction conditions employed.

**Scheme 12:**
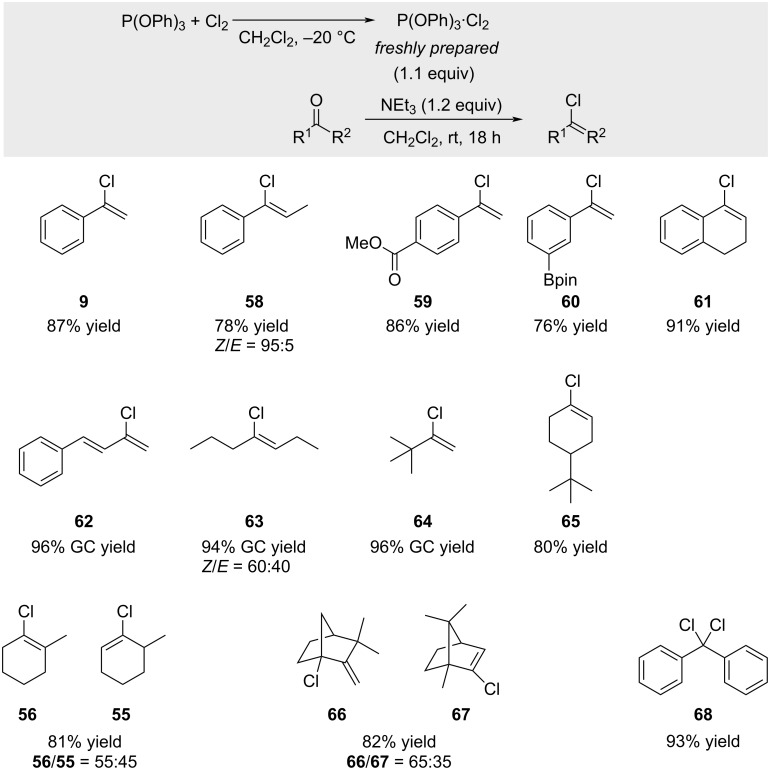
(PhO)_3_P–halogen-based reagents for the synthesis of alkenyl halides.

In 2007, Prati reported the use of (PhO)_3_P–halogen-based reagents for the synthesis of alkenyl halides (Scheme 12) [64]. A variety of alkenyl chlorides was obtained in good yields, with functional groups such as esters and boronates well tolerated. When camphor was treated with (PhO)_3_P·Cl_2_ (TPPCl_2_), the two constitutional isomeric chloro derivatives **66** and **67** were formed in a 65:35 ratio. In the case of non-enolizable ketones such as benzophenone, dichlorodiphenylmethane **68** was obtained in 93% yield. Aldehydes consistently afforded *gem*-dichlorides in excellent yields, regardless of enolizability (not shown). It is also worth noting that as early as 1959, Horner reported the synthesis of 1-chlorocyclohexene in 45% yield from cyclohexanone using PPh_3_^.^Cl_2_ complex (not shown) [65].

**Scheme 13:**
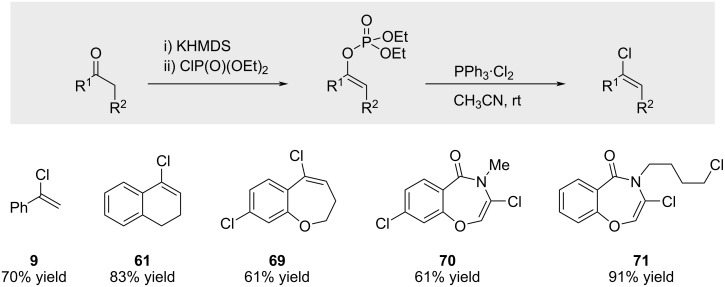
Preparation of alkenyl chlorides from alkenyl phosphates.

In 2005, Kamei reported that alkenyl chlorides could be efficiently prepared from the corresponding alkenyl phosphate intermediates via treatment with a triphenylphosphine–chlorine complex (Scheme 13) [66].

A major disadvantage of the Prati, Horner and Kamei procedures is that the active phosphorous species has to be freshly prepared by bubbling highly toxic, elemental chorine into solutions of P(OPh)_3_ or PPh_3_.

**Scheme 14:**
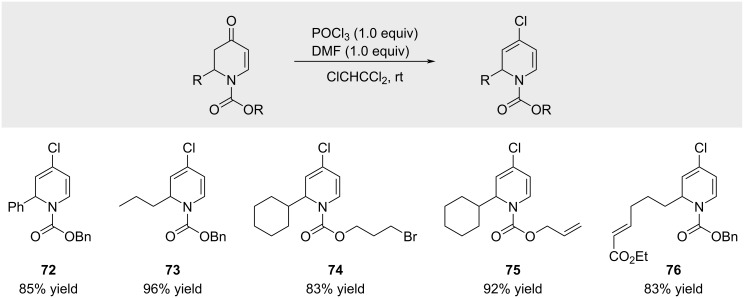
Preparation of alkenyl chlorides by treatment of ketones with the Vilsmeier reagent.

In 1993, Comins and co-workers reported the serendipitous discovery that *N*-acyl-2,3-dihydro-4-pyridinones react with the Vilsmeier reagent to afford the corresponding alkenyl chlorides (Scheme 14) [67]. Notably, dihydropyridones failed to react with POCl₃ in the absence of DMF.

**Scheme 15:**
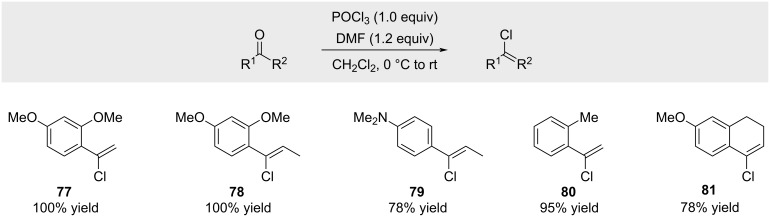
Preparation of electron-rich alkenyl chlorides by treatment of ketones with the Vilsmeier reagent.

In 2003, Wähälä reported a closely related reaction in which an in situ-generated Vilsmeier reagent converted aryl ketones bearing electron-donating substituents at the *ortho*- or *para*-position into alkenyl chlorides [68]. The reported yields corresponded to crude products and therefore do not reflect isolated yields of pure compounds (Scheme 15).

**Scheme 16:**
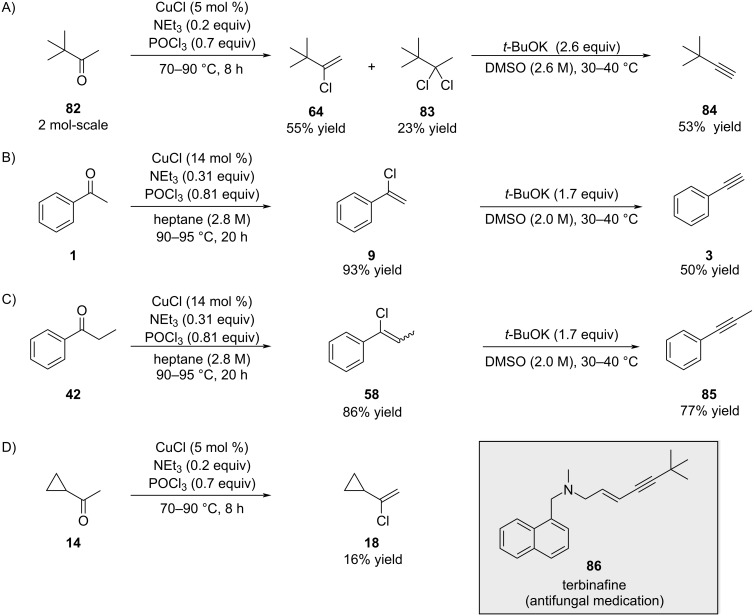
Cu-promoted synthesis of alkenyl chlorides from ketones and POCl_3_.

In 2006, Apotex reported the synthesis of alkenyl chlorides using POCl_3_ in the presence of triethylamine and a copper catalyst (Scheme 16) [69]. The preparation of compound **84** is of industrial significance due to its application in the synthesis of terbinafine (**86**), an antifungal agent.

**Figure 5:**
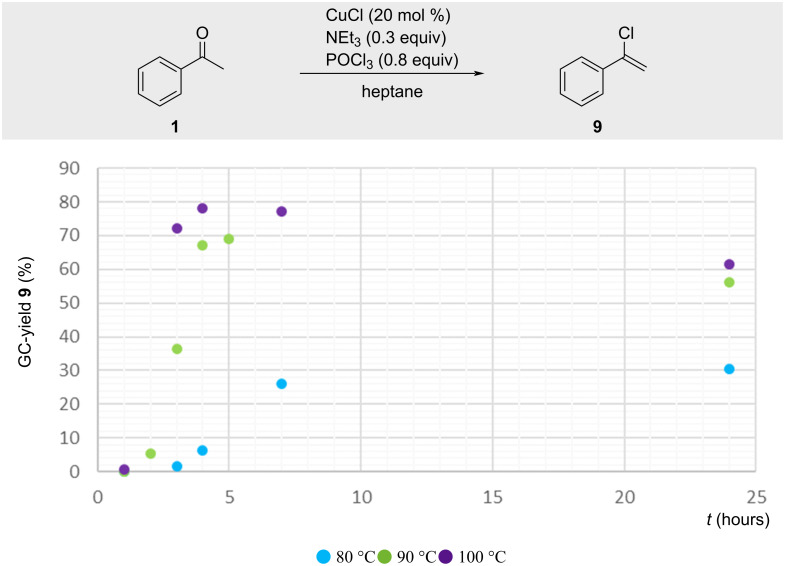
GC yield of **9** depending on time and reaction temperature.

The reaction attracted our interest, prompting a more detailed investigation. Kinetic studies with acetophenone revealed an induction period prior to reaction onset (Figure 5). Elevated temperatures shortened the initiation time, whereas lower temperatures extended it to several hours. Both temperature and reaction time significantly influenced the overall yield.

**Figure 6:**
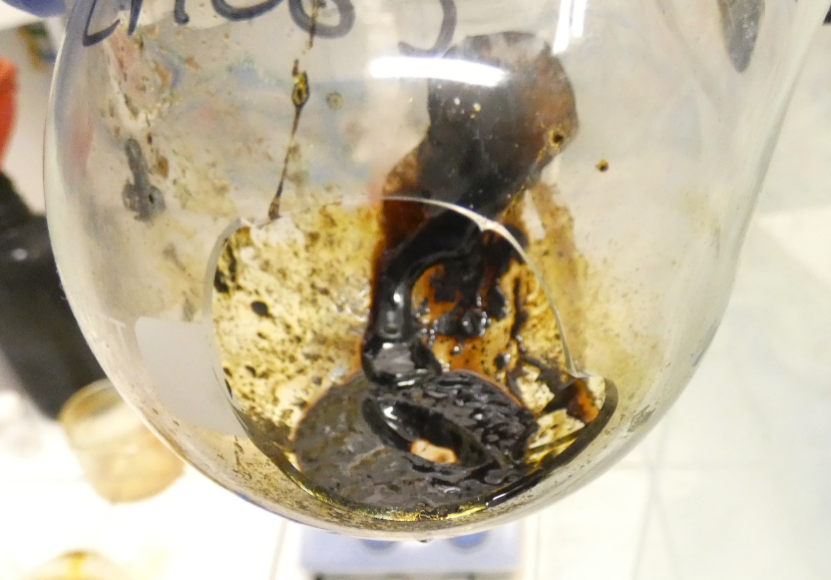
Broken reaction flask after attempts to clean the polymerized residue.

The decline in yield over time can potentially be attributed to polymerization side reactions of product **9** under the reaction conditions. Visual evidence of polymerization included difficulty in cleaning the reaction flask and the formation of solid polymer deposits that were irreversibly adhered (Figure 6, oval stirring bar left a mark in the solid polymer).

**Figure 7:**
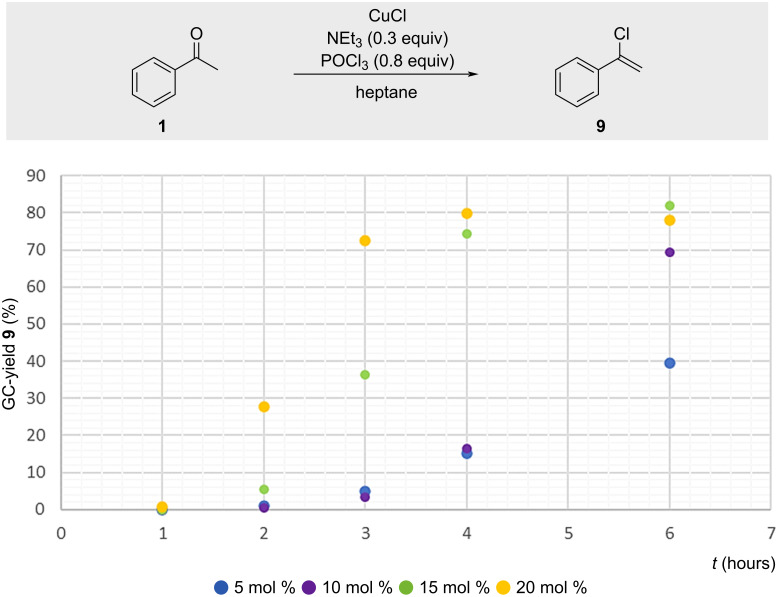
GC yield of **9** depending on the amount of CuCl and time.

Various catalyst loadings of CuCl (5–20 mol %) were evaluated, with 15–20 mol % required to achieve useful yields of **9** within several hours (Figure 7). Mechanistic analysis proved challenging, as the catalyst is generated in situ and likely present only in trace amounts. All our attempts to accelerate the reaction through addition of various mono- and bidentate phosphorus ligands were unsuccessful.

A similar trend was observed with NEt_3_, which afforded optimal yields at 20–30 mol % loading (not shown). Thus, the reaction is synthetically valuable but necessitates careful control of reagent quantities and reaction conditions for optimal yields.

**Scheme 17:**
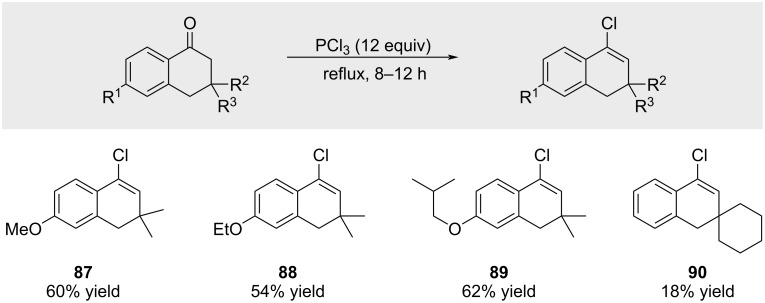
Treatment of 4-chromanones with PCl_3_.

Chemists from Alkaloida reported that 4-chromanones undergo transformation to the corresponding 4-halo-2*H*-chromenes upon treatment with PCl_3_ (Scheme 17) [70]. The reaction appears limited to electron-rich chromanones, as evidenced by decreased yields upon removal of the electron-donating ether substituent (e.g., compound **90**). The authors further noted that the products require cold storage due to their sensitivity.

**Scheme 18:**
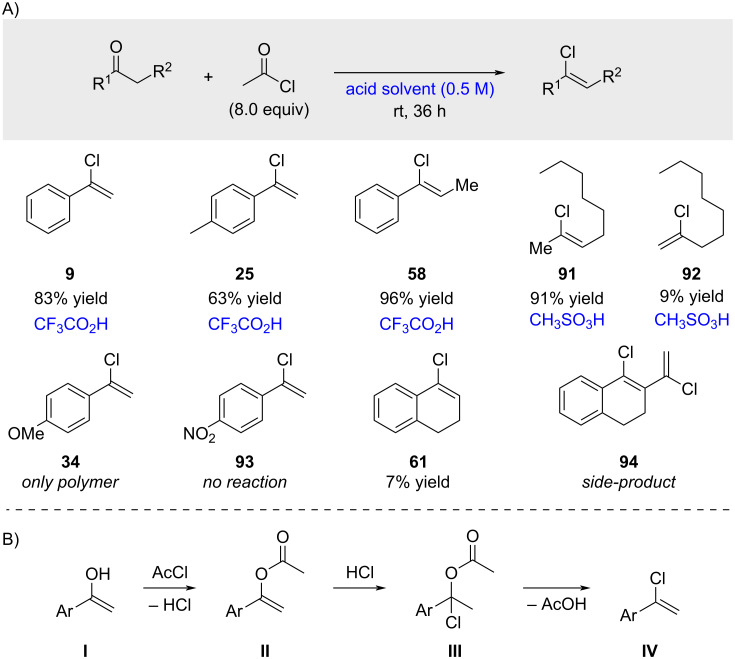
Synthesis of alkenyl chlorides from the reaction of ketones with acyl chlorides.

**Deoxychlorination with acyl chlorides:** The first synthesis of alkenyl chlorides from acyl chlorides was reported by Moughamir and Mestdagh in 1999 (Scheme 18A) [71]. They observed that strongly acidic solvents, including trifluoroacetic acid and methanesulfonic acid, provided the corresponding products in good yields with excellent stereoselectivity, exclusively yielding the *Z*-isomer. Nonan-2-one required activation with the stronger methanesulfonic acid and gave a 91:9 regioisomeric mixture of **91** to **92**. 1-Tetralone provided the corresponding product **61** in only 7% yield; the acid additive was not specified in this case. The authors were unable to efficiently separate **61** from side product **94**, which was likely formed via addition of acetyl chloride to **61** followed by conversion of the ketone to the alkenyl chloride. Regarding the mechanism (Scheme 18B), acid-catalyzed enolization of the ketone is proposed (enol I), followed by acetylation under the reaction conditions (acetate II). Subsequent addition of HCl to intermediate II generates chloride III, which undergoes elimination to yield the desired product IV.

**Scheme 19:**
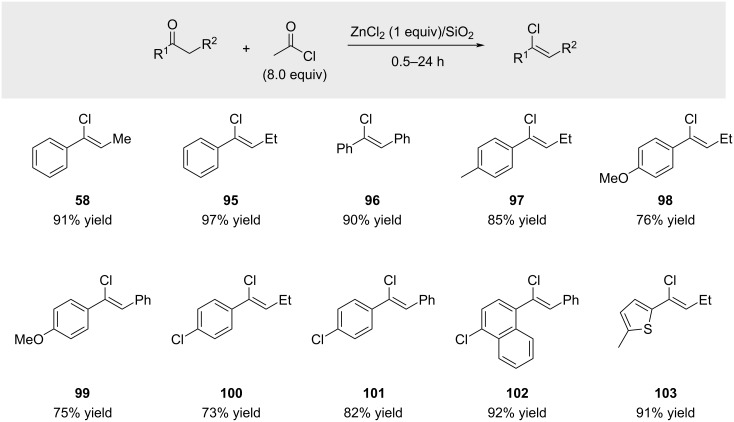
ZnCl_2_-promoted alkenyl chloride synthesis.

Kodomari and co-workers serendipitously discovered a related reaction in which a large excess of acetyl chloride (8 equivalents) in the presence of ZnCl₂ supported on silica gel converted ketones into the corresponding alkenyl chlorides (Scheme 19) [72]. The scope appears to be limited to aromatic ketones furnishing non-terminal alkenyl chlorides.

**Scheme 20:**
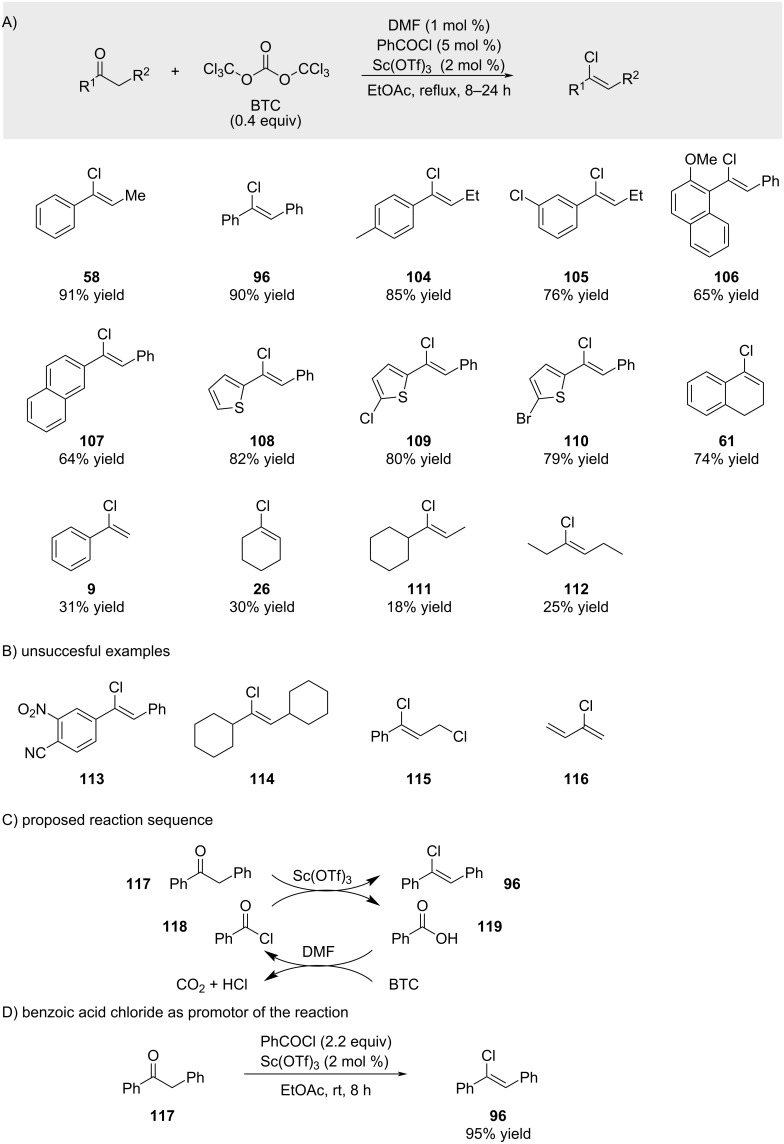
Regeneration of acid chlorides by triphosgene.

Su and co-workers reported the synthesis of alkenyl chlorides using a multicomponent reagent system (Scheme 20A). In an effort to render the transformation more environmentally benign, benzoyl chloride was replaced with triphosgene [73] [bis(trichloromethyl) carbonate, BTC], a putative “greener” surrogate [74]. Nevertheless, the high vapor pressure and associated safety concerns of BTC [75–76], represent a notable limitation. The substrate scope parallels that described by Kodomari (Scheme 19), though it includes one example of a terminal alkenyl chloride (compound **9**), obtained in modest yield. Several ketone substrates proved unreactive, affording no or only trace amounts of the desired products (Scheme 20B). The authors proposed that BTC functions by regenerating benzoyl chloride in situ from benzoic acid, thereby allowing the use of benzoyl chloride in catalytic quantities (Scheme 20C). Under similar conditions – benzoyl chloride (2.2 equiv), room temperature, absence of DMF – the alkenyl chloride **96** was isolated in 95% yield (Scheme 20D). The transformation is mechanistically and conceptually closely related to earlier findings reported by Moughamir and Mestdagh (see Scheme 18).

**Scheme 21:**
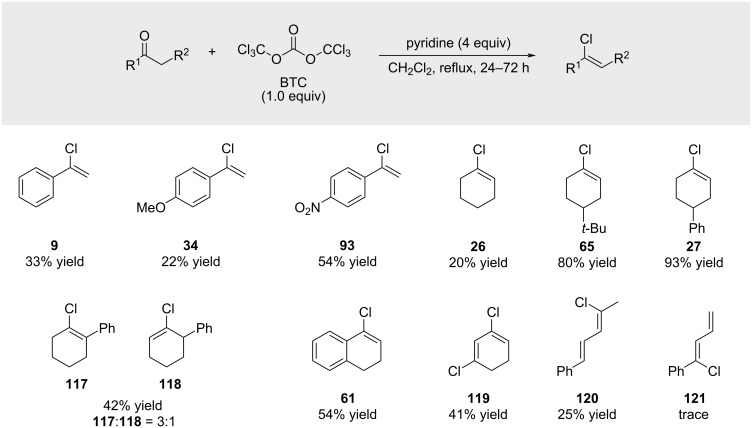
Alkenyl chlorides from ketones and triphosgene.

In 2015, Kartika and co-workers reported a metal and acid-free synthesis of alkenyl chlorides using triphosgene (BTC) (Scheme 21) [77]. In the presence of excess pyridine, BTC mediated the transformation of various ketones into the corresponding alkenyl chlorides, which were obtained in moderate isolated yields despite consistently high GC yields. The authors attributed this discrepancy to product degradation during purification on neutralized silica gel (1% NEt₃) or neutral alumina. However, this hypothesis was not experimentally verified, as no control experiment involving the passage of a pure, non-volatile alkenyl chloride through the purification media was reported to assess compound loss or degradation at the purification step.

#### 1.2 Exchange reactions

This chapter deals with exchange reactions, meaning that a given substituent on the alkene is formally exchanged for chlorine. The reactions are very different from each other in terms of mechanism and for a full discussion the review by Evano should be consulted [37].

**Scheme 22:**
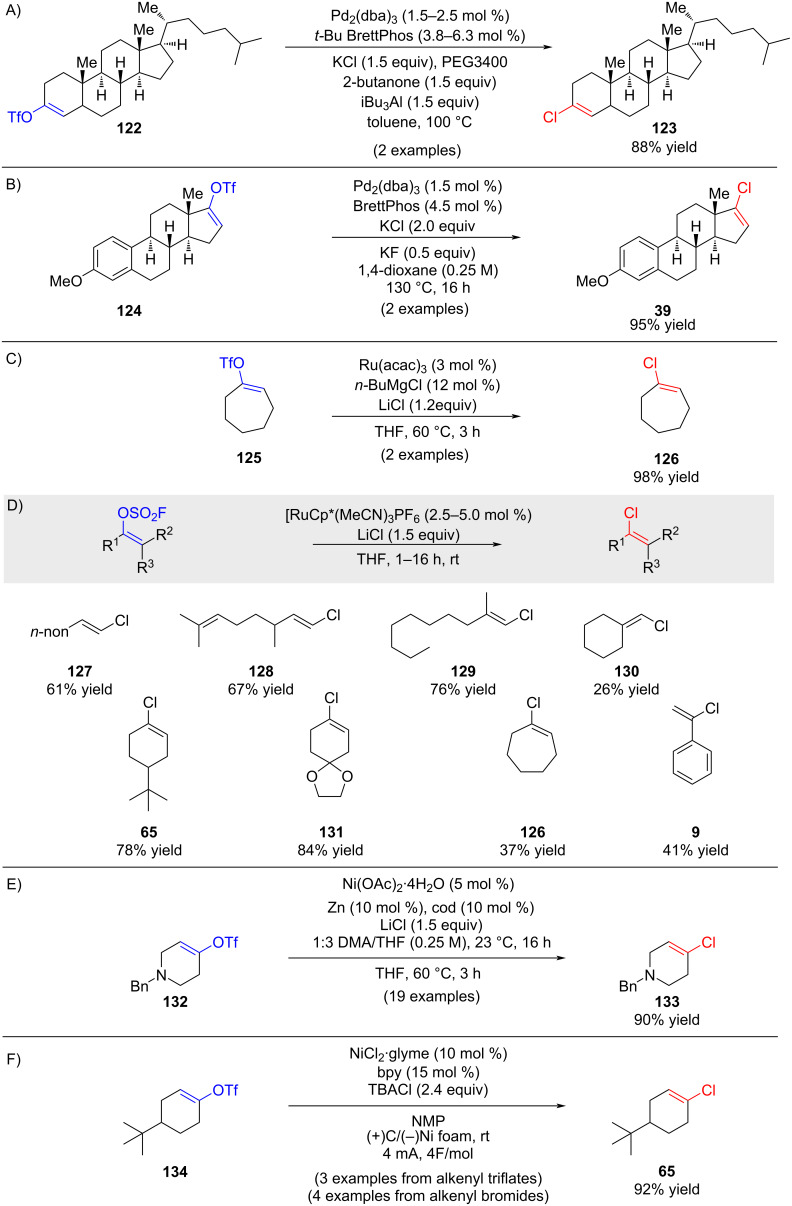
Various substitution reactions.

In 2010, Buchwald and co-workers reported a palladium-catalyzed transformation of vinyl triflates to alkenyl chlorides (Scheme 22A) [78]. An improved variant of this methodology was published the following year (Scheme 22B) [79]. Independently, in 2009, Hayashi described a ruthenium-catalyzed conversion of alkenyl triflates to alkenyl chlorides (Scheme 22C) [80]. A subsequent study from the same group demonstrated that [Cp*Ru(MeCN)_3_]OTf could serve as an alternative catalyst, thereby avoiding the need for in situ reduction of Ru(III) to Ru(II) by organometallic reagents such as Grignards (not shown) [81]. More recently, a collaborative study between Bayer and researchers at the University of Strasbourg disclosed a ruthenium-catalyzed halide exchange of vinyl fluorosulfonates, which were prepared from ketones and sulfuryl fluoride (SO_2_F_2_) in the presence of DBU (1,8-diazabicyclo[5.4.0]undec-7-ene) (Scheme 22D) [82]. Complementary to this, Reisman and co-workers reported a nickel-catalyzed conversion of vinyl triflates to alkenyl chlorides in 2019 (Scheme 22E) [83]. In 2024, Payard, Perrin, and Vantourout introduced an electrochemically driven nickel-catalyzed halide exchange, enabling the conversion of alkenyl bromides and triflates to the corresponding chlorides using tetrabutylammonium chloride (TBACl) as the chloride source (Scheme 22F) [84].

**Scheme 23:**
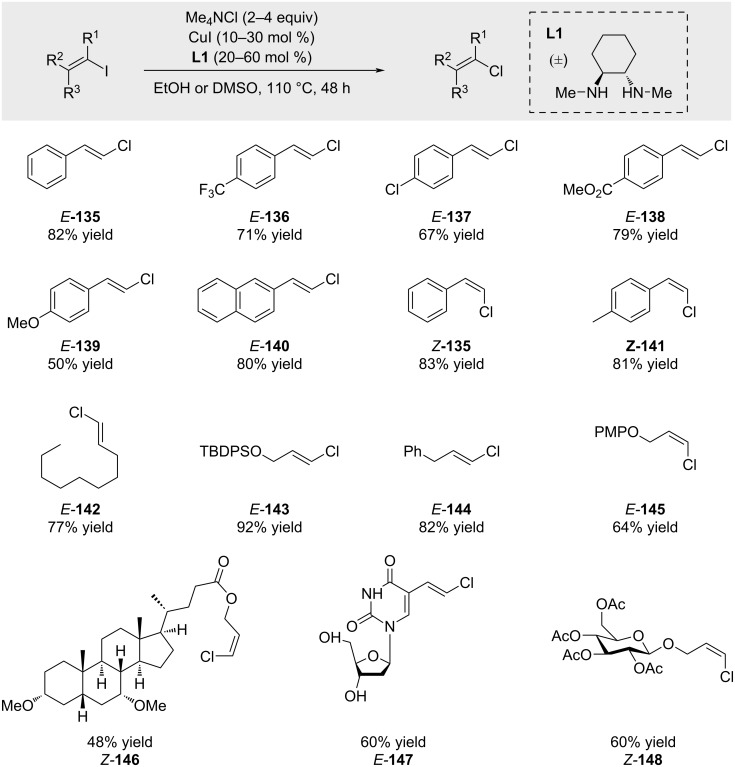
Vinylic Finkelstein reactions reported by Evano and co-workers.

A fundamentally different approach was developed by Evano and co-workers [85–86], who reported a vinylic Finkelstein-type halide exchange reaction (Scheme 23). This strategy leverages the decreasing bond-dissociation energies across the halogen series – C–Cl (*D*_298_(C−Cl) = 395 kJ mol^−1^) and C–Br (*D*_298_(C–Br) = 318 kJ mol^−1^) bonds compared to the C–I bond (*D*_298_(C–I) = 253 kJ mol^−1^) to drive the substitution of iodide or bromide by chloride. In the presence of a copper catalyst and tetramethylammonium chloride (Me_4_NCl) as the chloride source, alkenyl iodides and bromides were efficiently converted to the corresponding alkenyl chlorides. Importantly, the transformation proceeded with full retention of the double bond geometry. For a comprehensive discussion, the reader is referred to the Evano’s full account [37].

#### 1.3 Hydrochlorination

One of the most widely produced alkenyl chlorides is vinyl chloride. As of 2025, its global annual production exceeds 50 million tons, driven almost exclusively by its use as the monomer for polyvinyl chloride (PVC) [87]. The hydrochlorination of acetylene remains a key industrial process for vinyl chloride synthesis – particularly in China, where approximately 70% of the total production is based on this route. The reaction is typically catalyzed by carbon-supported mercuric chloride which is a major environmental concern [88].

Due to the extensive body of literature and the large number of patents related to the hydrochlorination of acetylene, this area represents a distinct and specialized field within hydrochlorination chemistry. As such, it is not covered in this review, and the reader is referred to the recent comprehensive accounts by Li [89] and Mitchenko [90] for further details.

**Scheme 24:**
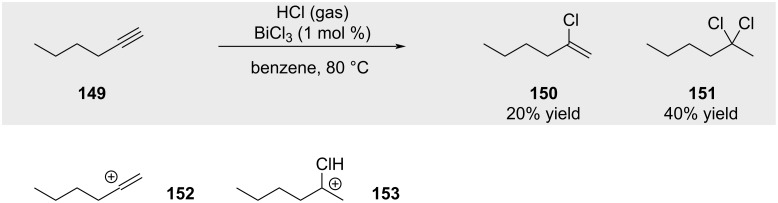
Challenge of selective monohydrochlorination of alkynes.

Hydrochlorination of terminal alkynes frequently results in complex product mixtures due to the greater reactivity of the initially formed alkenyl chloride intermediate (e.g., **150**, Scheme 24) towards further protonation – often proceeding via more stabilized tertiary carbocation intermediates (for example: **153** is more stable compared to initially formed alkenyl cation **152**, Scheme 24) [91]. This protonation pathway commonly leads to the formation of gem-dichloride species **151**, which can subsequently undergo hydrolysis in the presence of trace water to furnish the corresponding ketone (see Scheme 27, formation of ketone **167**) [92].

**Scheme 25:**
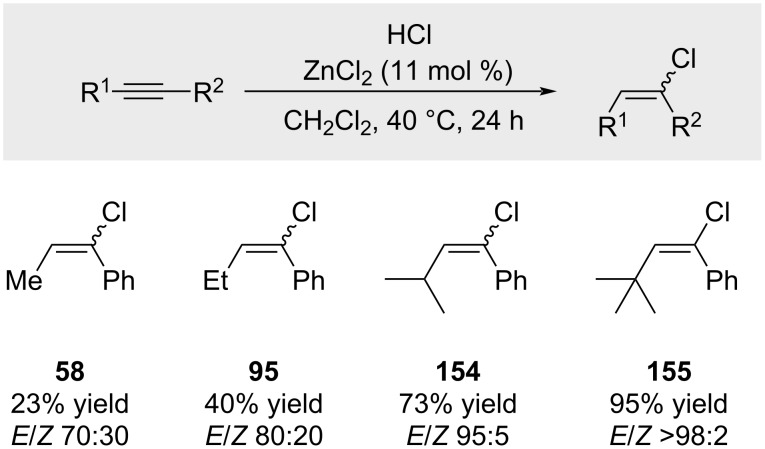
Sterically encumbered internal alkynes furnish the hydrochlorination products in high yield.

Furthermore, Melloni were the first to demonstrate that highly hindered internal alkynes resist double HCl addition (Scheme 25) [93]. In cases where a quaternary substituent is present (compound **155**), the reaction proceeds with excellent yields.

**Scheme 26:**
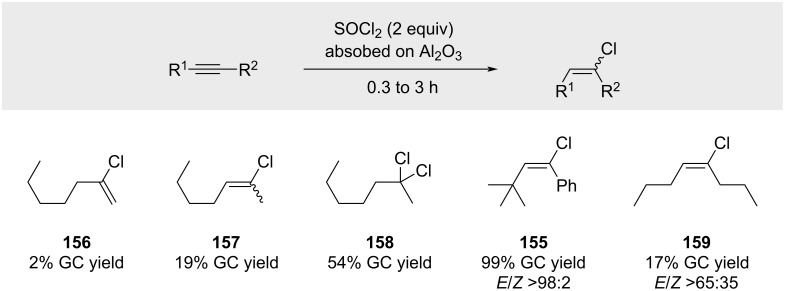
Recent work by Kropp with HCl absorbed on alumina.

A more recent study by Kropp on surface-mediated hydrochlorination reactions (Scheme 26) [94] also demonstrated that hydrochlorination of 1-heptyne yields a mixture of three products **156**, **157**, and **158**.

The target alkenyl chloride **156** was isolated only in trace amounts, alongside isomerized alkene **157** and dichloride **158**. Notably, only the sterically hindered *tert*-butyl-substituted alkyne afforded the desired product **155** in high yields, whereas hydrochlorination of 4-octyne proved inefficient to deliver compound **159**.

**Scheme 27:**
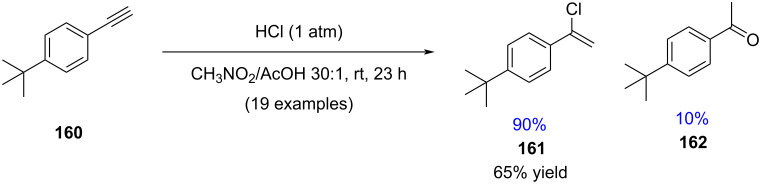
High selectivities for monhydrochlorination with nitromethane/acetic acid as solvent.

Thus, the hydrochlorination of terminal aliphatic alkynes serves as a valuable benchmark reaction to differentiate between purely ionic and transition-metal-catalyzed hydrochlorination processes which often show highly monoselective reactions [95]. Somewhat surprisingly, a recent study by Dai demonstrated that aromatic alkynes can undergo hydrochlorination with good selectivity for the mono-addition when a nitromethane/acetic acid solvent mixture is employed (Scheme 27) [96].

**Figure 8:**
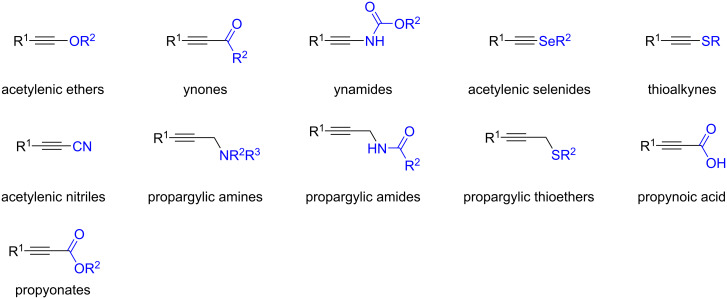
Functionalized alkynes which typically afford the monhydrochlorinated products.

In contrast, selective monohydrochlorination is commonly observed for alkynes bearing coordinating substituents such as acetylenic ethers [97–98], ynones [99], ynamides [98–102], acetylenic selenides [103], thioalkynes [98,104], acetylenic nitriles [105], propargyl amines [106], propargylic amides [107], propargylic thioethers [106], propynoic acids [105], and propynoates [105,108] (Figure 8).

Over the past two decades, considerable efforts have been devoted to the selective monohydrochlorination of alkynes, particularly through the use of transition-metal catalysis. Comprehensive reviews by Lu [40] and Nishiwaki [41] have extensively covered these advances.

**Scheme 28:**
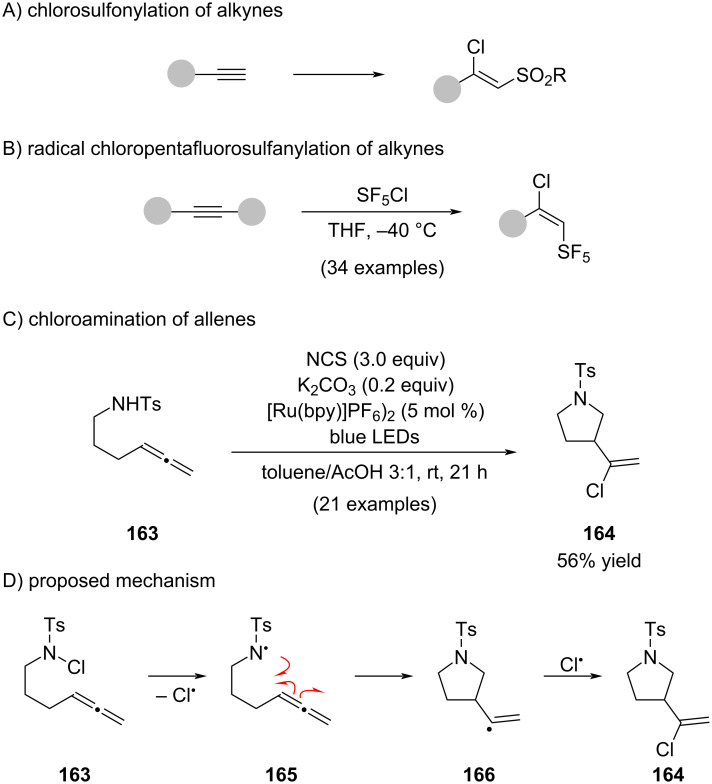
Related chorosulfonylation and chloroamination reactions.

We also want to briefly mention the work on chlorosulfonylation [109–111] (Scheme 28A) and chloropentafluorosulfanylation of alkynes (Scheme 28B) [112]. Chloroaminations of alkynes are well known and have been summarized in recent work by Renzi concerning the chloroamination of allenes. (Scheme 28C) [113]. A simplified mechanistic proposal is shown in Scheme 28D, involving initial generation of a nitrogen-centered radical **165**, which undergoes intramolecular addition to the pendant allene to form a vinyl radical intermediate **166**. Subsequent interception of **166** by an N–Cl bond furnishes alkenyl chloride **164**.

#### 1.4 Reaction of alkenyl metals with chlorine electrophiles

**Scheme 29:**
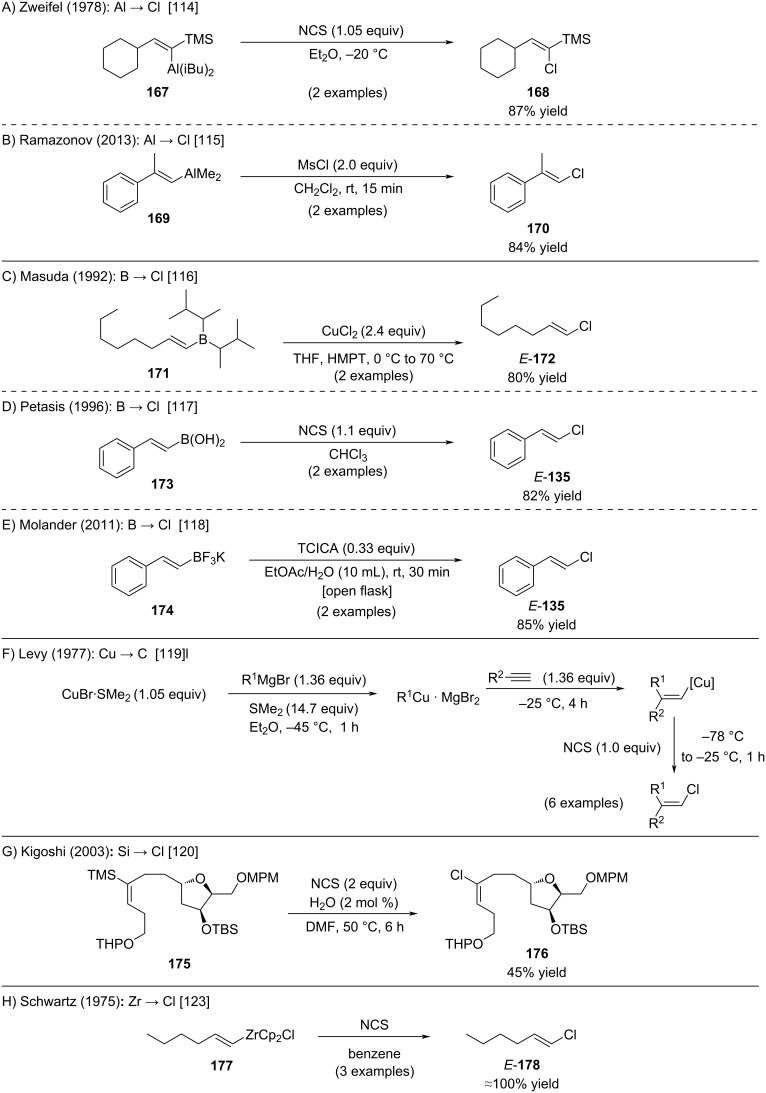
Reaction of organometallic reagents with chlorine electrophiles.

A wide range of alkenylmetal species has been employed for the synthesis of alkenyl chlorides via reaction with chlorine electrophiles (Scheme 29). In a study from 1978, Zweifel demonstrated that 1,1-silyl(aluminum)alkenyl intermediates (e.g., **167**) undergo highly selective chlorination at the aluminum-bound site using *N*-chlorosuccinimide (NCS) (Scheme 29A) [114]. Decades later, Ramazanov expanded this approach, showing that alkenylaluminums (e.g., **169**) derived from carboalumination react efficiently with mesityl chloride (MsCl) to furnish alkenyl chloride **170** (Scheme 29B) [115]. In contrast, analogous hydroalumination products obtained from hydroalumination of alkynes with DIBAL-H gave only trace amounts of the desired chlorinated products (not shown). Chlorodeboronation offers another powerful entry to alkenyl chlorides (Scheme 29C–E). Masuda [116], Petasis [117], and Molander [118] have independently demonstrated that organoboranes, alkenylboronic acids, and trifluoroborates undergo CuCl_2_-, NCS-, or trichloroisocyanuric acid (TCICA)-mediated chlorination to furnish alkenyl chlorides. Earlier, Levy showed that alkenylcopper reagents could be selectively converted to chlorinated products with excellent stereochemical control using NCS (Scheme 29F) [119]. Kigoshi reported the reaction of alkenylsilane with NCS to afford compound **176** which served as building block for the total synthesis of 15-*epi*-haterumalide NA (Scheme 29G) [120]. Interestingly, the transformation of alkenylsilanes to alkenyl chlorides was also utilized in the total synthesis of (*S*)-jamaicamide C and laingolide B (not shown) [121–122]. Schwartz’s work on alkenylzirconium complexes revealed high-yielding, stereoretentive transformations under similar conditions (Scheme 29H) [123]. In a complementary study, Srebnik showed that bimetallic 1,1-alkenyl species bearing both boron and zirconium centers selectively undergo chlorination at the zirconium site, leaving the boron moiety untouched (not shown) [124].

#### 1.5 Elimination reactions

**Scheme 30:**
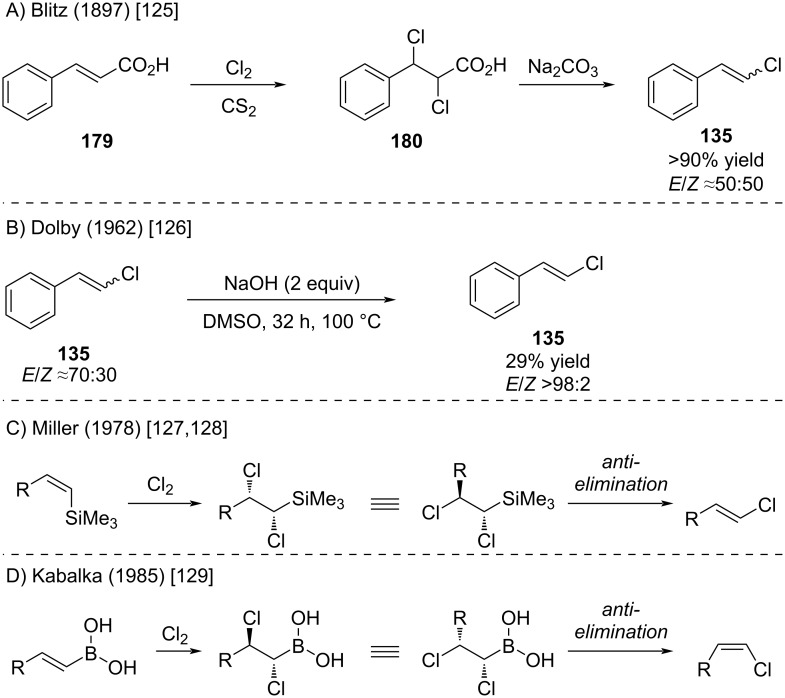
Elimination reactions of dichlorides to furnish alkenyl chlorides.

The first elimination-based synthesis of alkenyl chlorides was reported by Biltz as early as 1897 (Scheme 30A) [125]. Decades later, Dolby demonstrated that a 70:30 *E*/*Z* mixture of precursors could be selectively converted into the pure *E*-alkenyl chloride **135** by treatment with NaOH in DMSO (Scheme 30B) [126]. Related strategies involving the addition of chlorine to alkenylsilanes or alkenylboranes, followed by base-induced elimination, were developed by Miller [127–128] and Kabalka [129] (Scheme 30C and D). In these cases, elimination was effected by fluoride or alkoxide, resulting in the loss of the boryl or silyl moiety and formation of the desired alkenyl chloride.

**Scheme 31:**
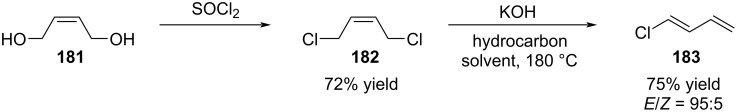
Elimination reactions of allyl chloride **182** to furnish alkenyl chloride **183**.

Alexakis, Normant, and Fugier reported the synthesis of conjugated alkenyl chloride **183** by chlorination of *cis*-diol **181** and subsequent elimination of product **182** with KOH (Scheme 31) [130].

**Scheme 32:**
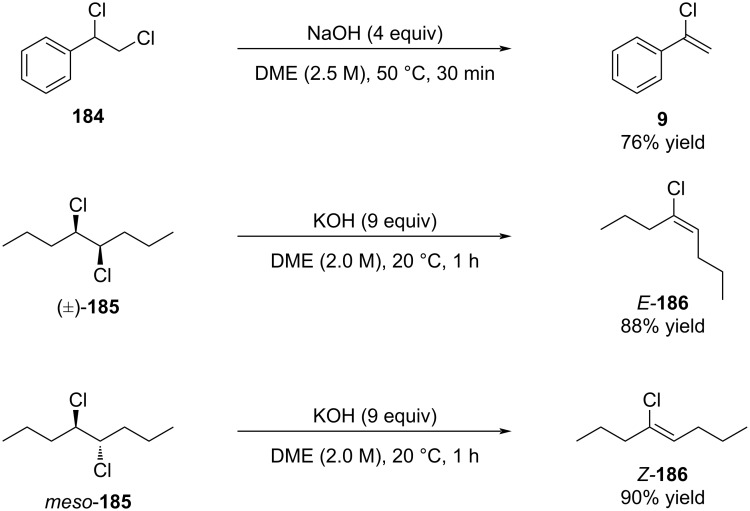
Detailed studies by Schlosser on the elimination of dichloro compounds.

Schlosser reported that treatment of 1,2-dichloroalkanes with sodium or potassium hydroxide in dimethoxyethane effected selective elimination, furnishing the corresponding alkenyl chlorides in useful yields (Scheme 32) [131].

**Scheme 33:**
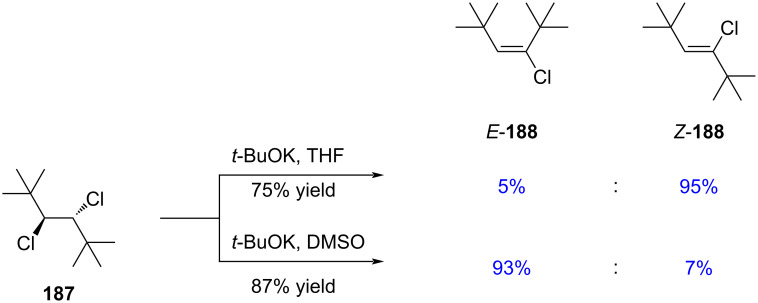
Stereoselective variation caused by change of solvent.

Furthermore, he demonstrated that the stereochemical outcome is strongly influenced by the choice of solvent (Scheme 33) [132].

**Scheme 34:**
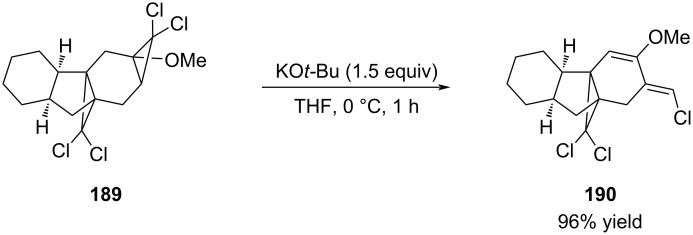
Elimination of *gem*-dichloride **189** to afford alkene **190**.

An interesting elimination of a dichlorocyclopropane was reported by Banwell in the context of the synthesis of tetracyclic frameworks related to gibberellins (Scheme 34) [133]. The authors do not propose a mechanism for this reaction. A related reaction was recently reported by Greatrex and co-worker (not shown) [134].

**Scheme 35:**
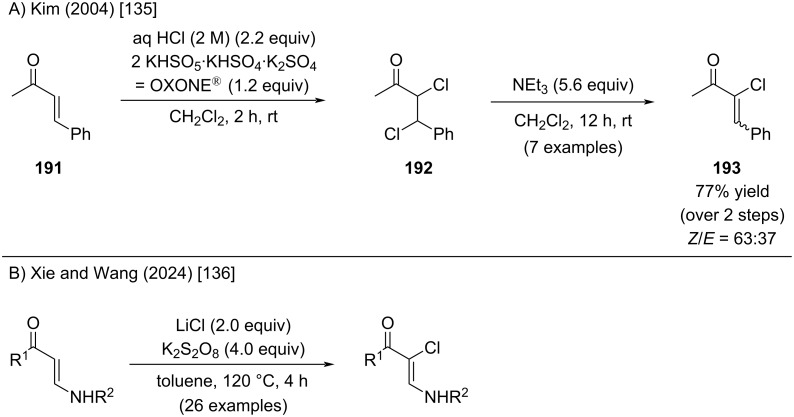
Oxidation of enones to dichlorides and in situ elimination thereof.

Kim and co-workers reported a convenient α-chlorination of enones mediated by in situ oxidation of HCl with oxone (potassium peroxymonosulfate) to generate molecular chlorine (Scheme 35A) [135]. Electrophilic addition of chlorine across the enone double bond, followed by triethylamine-induced elimination, furnished the corresponding alkenyl chlorides (e.g., compound **193**) in good yields. A similar procedure, employing K_2_S_2_O_8_ as the oxidant, was recently reported by Wang and Yu (Scheme 35B) [136]. In their approach, the enamine substrate neutralizes the generated HCl, therefore eliminating the need for an external base such as NEt₃.

**Scheme 36:**
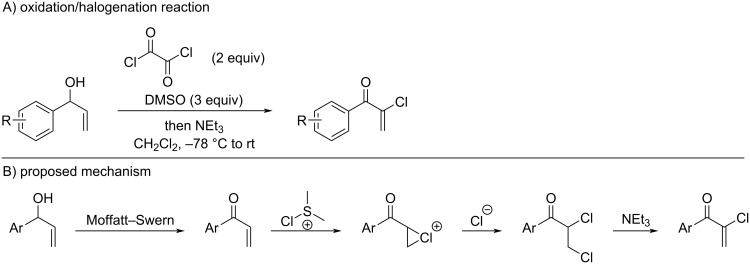
Oxidation of allylic alcohols to dichlorides and in situ elimination thereof.

Similar products can also be accessed via a tandem oxidation/halogenation reaction of allylic alcohols, as recently reported by Chisholm and co-workers (Scheme 36) [137]. Their approach involves oxidation of the allylic alcohol under Moffatt–Swern conditions, followed by halogenation of the resulting enone to generate a chloronium ion. Subsequent ring opening by chloride furnishes the corresponding dichloride, which eliminates HCl in the presence of triethylamine to afford the desired chloroenone in good yields.

**Scheme 37:**
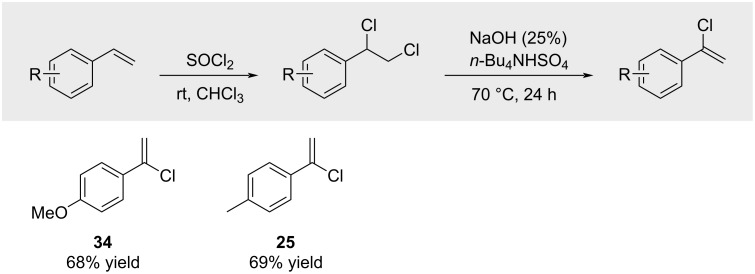
Chlorination of styrenes with SOCl_2_ and elimination thereof.

A recent report by Xiao also mentions the synthesis of dichlorides and their subsequent elimination to furnish alkenyl chlorides **34** and **25** (Scheme 37). However, no experimental details for these transformations are provided in the supporting information [138].

**Scheme 38:**
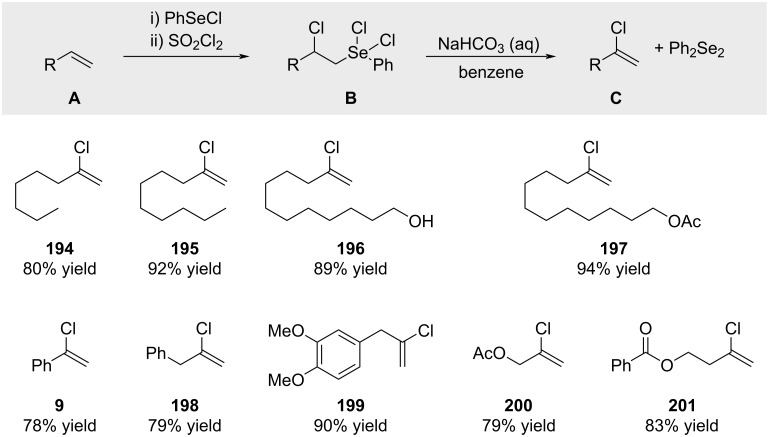
Chlorination of styrenes with SOCl_2_ and elimination thereof.

Engman reported the synthesis of alkenyl chlorides in a regiocontrolled way from terminal olefins by a sequence involving addition of PhSeCl, chlorination of the resulting selenides with SOCl_2_, and treatment with base (Scheme 38; yields refer to the transformation of **B** → **C**) [139].

**Scheme 39:**
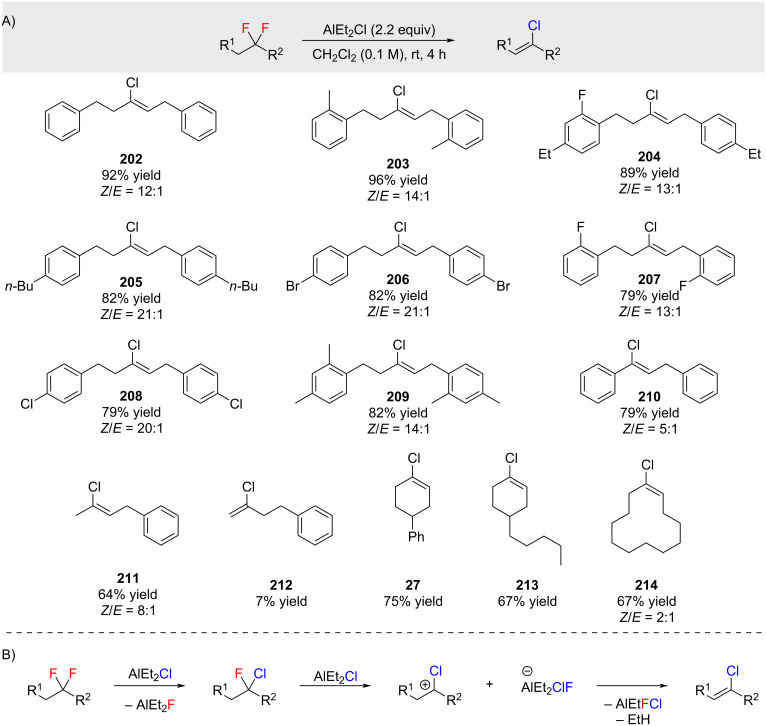
Fluorine–chlorine exchange followed by elimination.

A reaction which includes a fluorine–chlorine exchange and subsequent elimination of HCl was reported by Shibata and co-workers (Scheme 39) [140]. In this reaction the AlEt_2_Cl has a dual role of fluorine–chlorine exchange reagent and as a base.

Similar exchange reactions with AlCl_3_ and 1,1,1-trifluoroalkanones were recently reported by McLeod and co-workers (not shown) [141].

#### 1.6 Electrophilic additions to alkynes

**Scheme 40:**
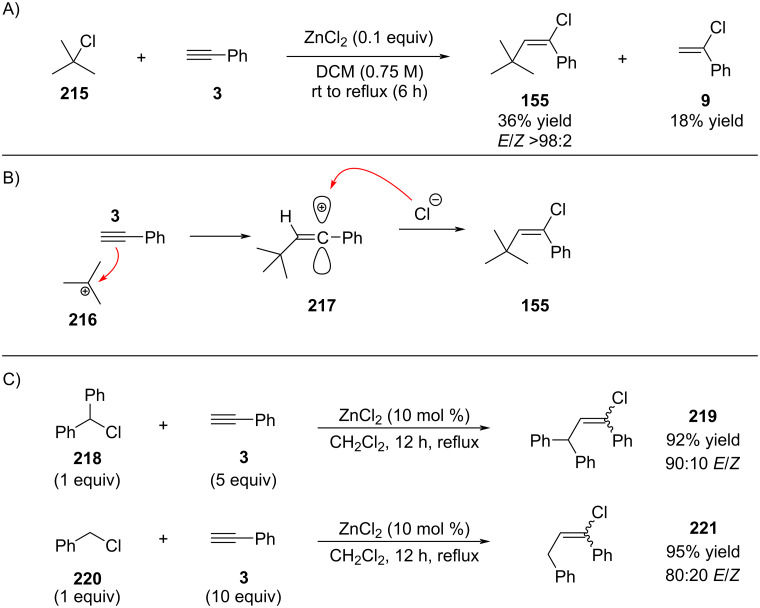
Intercepting cations with alkynes and trapping of the alkenyl cation intermediate with chloride.

Melloni was the first to report the addition of alkynes to in situ-generated tertiary aliphatic carbocations (Scheme 40A) [142]. Treatment of *tert*-butyl chloride (**215**) with alkyne **3** in the presence of ZnCl_2_ furnished alkenyl chlorides **155** and **9**, along with considerable amounts of polymeric byproducts [93,143]. Compound **155** was obtained with excellent stereoselectivity (>98:2 *E*/*Z*). Importantly, a lower alkyne-to-chloride ratio resulted in diminished yields of **155**.

The authors proposed that the key intermediate is a linear vinyl cation with a planar geometry (**217**, Scheme 40B), wherein the empty p-orbital lies in the molecular plane. As such, nucleophilic attack occurs within this plane, aligned with the axis of the vacant orbital. In the case depicted in Scheme 40B, the face *syn* to the hydrogen is significantly more accessible than the face *syn* to the bulky *tert*-butyl group, accounting for the high *E*-selectivity observed. In contrast, alkyl groups with reduced steric demand led to mixtures of *E*- and *Z*-isomers (Scheme 40C). Notably, in these less-selective cases, excess alkyne was required to achieve synthetically useful yields. For example, compound **221** was obtained in only 15% yield when phenylacetylene and benzyl chloride were employed in a 1:1 molar ratio.

**Scheme 41:**
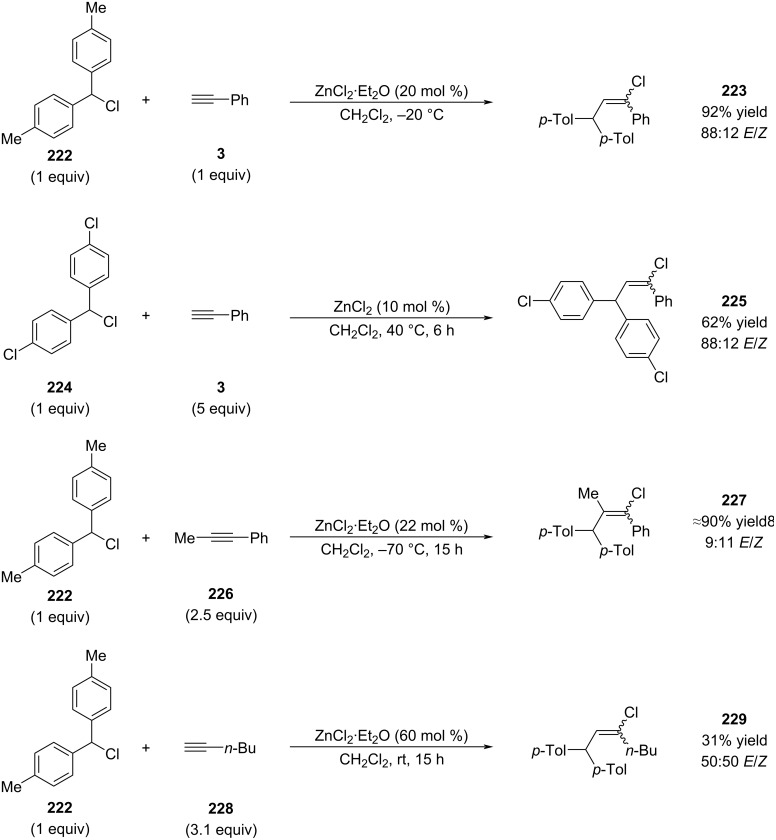
Investigations by Mayr and co-workers.

Mayr and co-workers reported a closely related transformation building on the earlier work by Melloni (Scheme 41) [144]. The final example in their study (compound **229**) illustrates that reactions involving aliphatic alkynes proceed with low efficiency and poor yields under these conditions. Notably, Mayr demonstrated that ZnCl_2_·etherate exhibits superior catalytic activity compared to anhydrous ZnCl_2_. Although counterintuitive from the standpoint of Lewis acidity, this enhanced reactivity was attributed to the significantly improved solubility of the etherate complex, which compensates for its diminished intrinsic Lewis acidity [145].

**Scheme 42:**
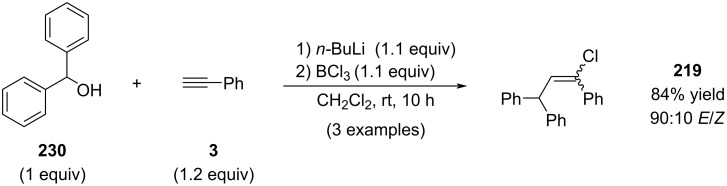
In situ activation of benzyl alcohol **230** with BCl_3_.

This transformation remained unexplored for nearly a decade until Kabalka demonstrated that the benzylic cation could be accessed directly from the corresponding alcohol via its conversion to the benzyloxyboron dichloride (Scheme 42) [146].

**Scheme 43:**
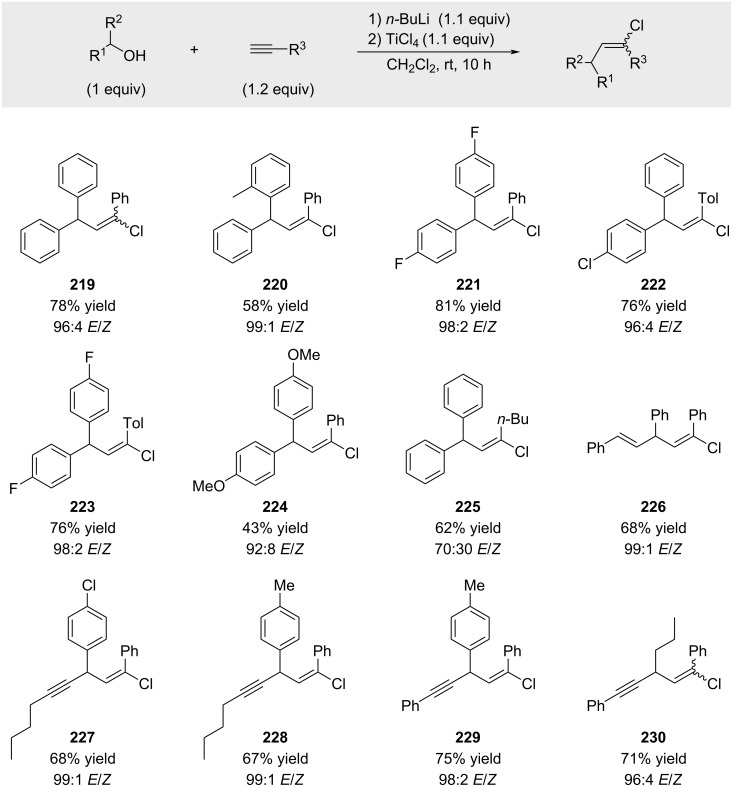
In situ activation of benzylic alcohols with TiCl_4_.

Subsequently, Kabalka reported an improved variant of the reaction in which TiCl_4_ was employed in place of BCl_3_ (Scheme 43) [147]. Interestingly, this modification led to an improved *E*/*Z* ratio compared to the values reported by Melloni (compare selectivity for compound **219** in Scheme 38 and Scheme 41). The enhanced selectivity is likely attributable to the increased steric demand of the TiCl_5_^−^ counterion, which may impose greater steric interactions during the critical chloride addition step [148]. The high reported yield of product **225** is unexpected. Examination of the corresponding ^1^H NMR spectrum revealed unusually broad signals, suggesting the possible presence of oligomeric or polymeric material in the isolated sample.

**Scheme 44:**
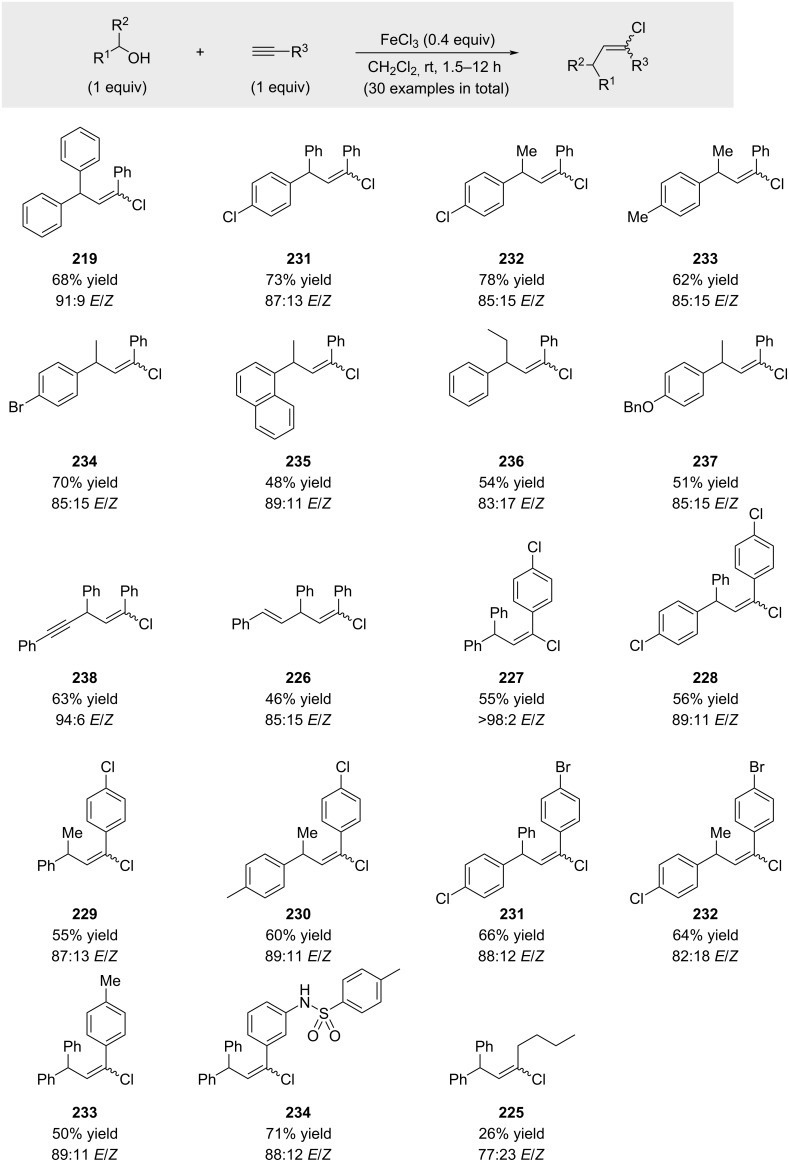
In situ activation of benzylic alcohols with FeCl_3_.

Biswas reported that benzylic, allylic, and propargylic alcohols could be activated by the addition of 0.4 equivalents of iron(III) chloride (Scheme 44) [149]. Although 19 examples were disclosed, limited information is available regarding functional group tolerance beyond an *N*-tosylated substrate to synthesize **234**. Consistent with earlier observations, aliphatic alkynes performed poorly under these conditions (compound **225**). One year later, Wang described a similar protocol employing 0.33 equivalents of FeCl_3_ with the principal difference being the use of 1,2-dibromoethane as solvent [150]. The reported substrate scope was comparatively narrow (not shown).

**Scheme 45:**
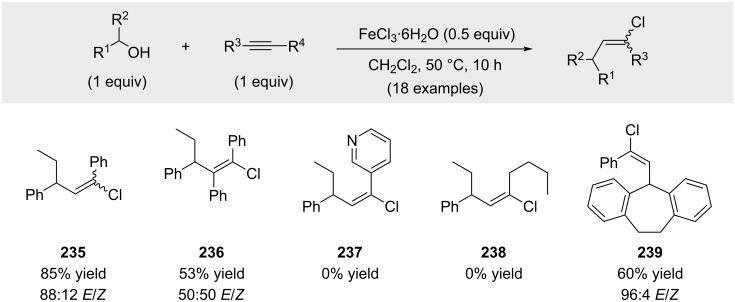
In situ activation of benzylic alcohols with FeCl_3_.

Simultaneously, Liu demonstrated that FeCl_3_·6H_2_O could also serve as an effective Lewis acid when the reaction temperature was raised to 50 °C (Scheme 45) [151]. As in previous studies, aliphatic alkynes were found to be inefficient coupling partners under these conditions (compound **238**).

**Scheme 46:**
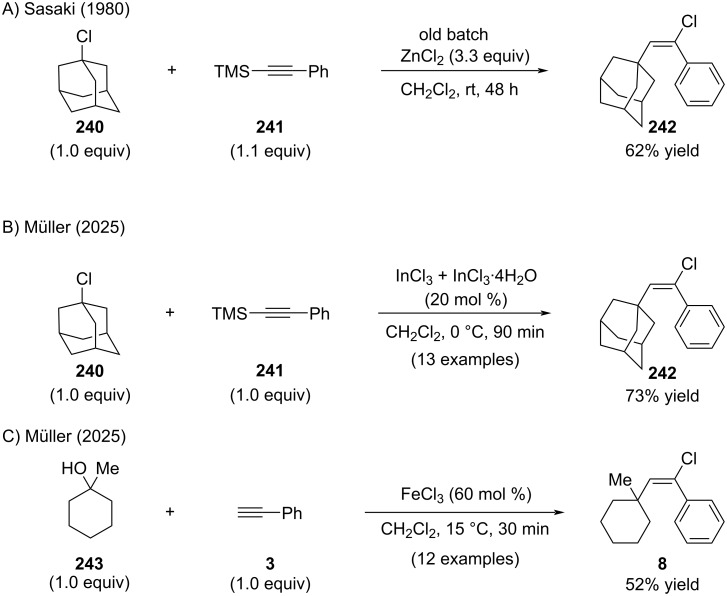
In situ activation of aliphatic chlorides and alcohols with ZnCl_2_, InCl_3_, and FeCl_3_.

Sasaki observed that hydrated zinc dichloride enabled the formation of alkenyl chloride **242** upon reaction of silylated alkyne **241** with 1-adamantyl chloride (**240**) (Scheme 46A) [152]. Revisiting this transformation, we found that hydrated indium trichloride afforded superior results under otherwise comparable conditions (Scheme 46B) [153]. In addition, we demonstrated that free alcohols could be employed as precursors using stoichiometric amounts of FeCl_3_ (Scheme 46C) [153]. In both cases, only tertiary aliphatic substrates were examined, generating non-stabilized carbocations. While the transformations proceeded cleanly, yields were generally low and the scope was restricted to non-functionalized arylalkynes.

**Scheme 47:**
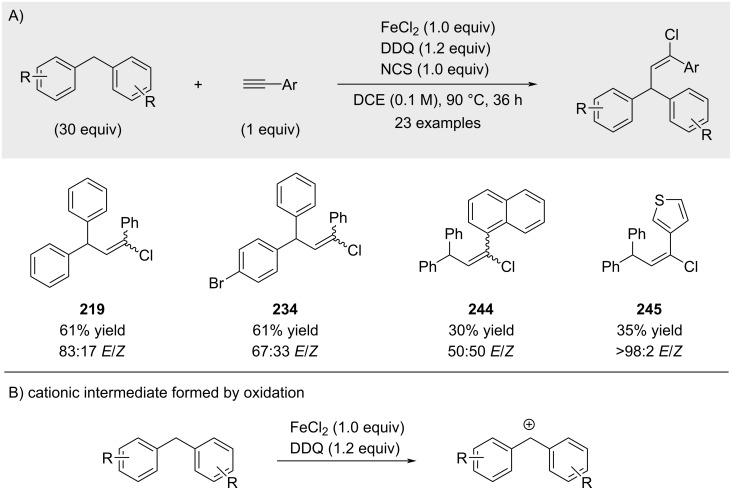
In situ generation of benzylic cations and trapping thereof with alkynes.

A one-pot procedure for the in situ generation of benzylic cations from hydrocarbon precursors using DDQ, followed by interception with alkynes, was reported by Shi and co-workers (Scheme 47) [154]. However, the authors did not clarify whether the corresponding benzyl chloride was formed in the absence of the alkyne trapping agent (trapping of cationic intermediate by chloride anion).

**Scheme 48:**
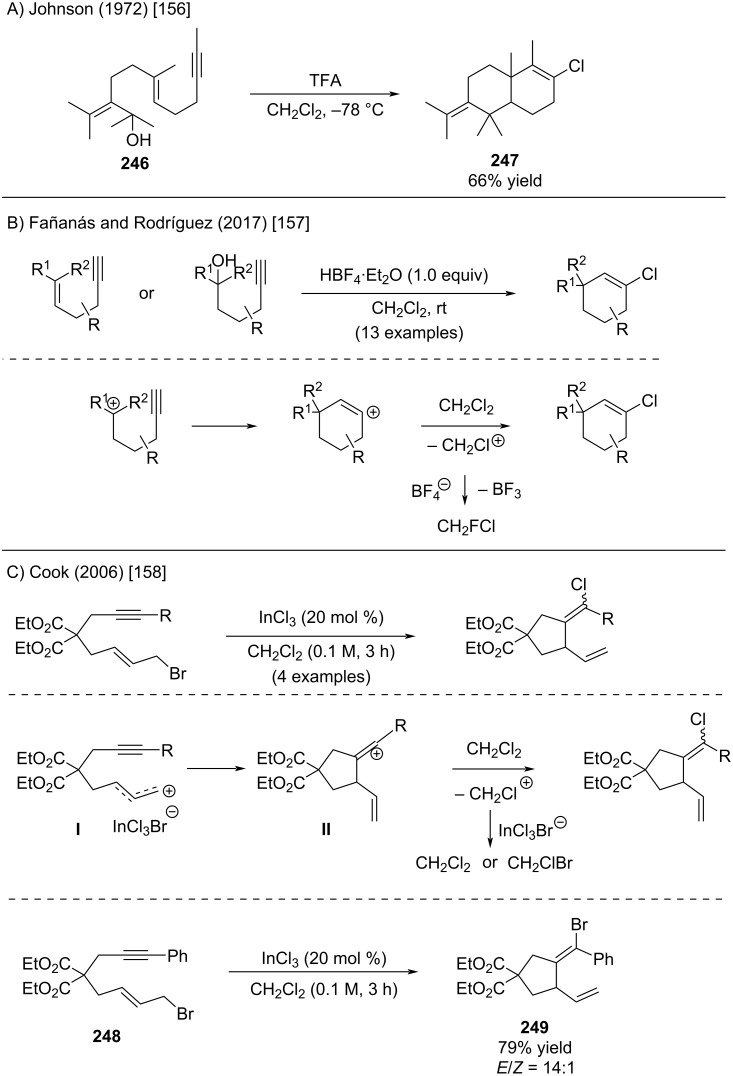
Intramolecular trapping reactions affording alkenyl halides.

Generation of tertiary aliphatic cations and intramolecular trapping with alkenes or alkynes is well known in the context of polyene cyclizations [155]. Building on Johnson’s earlier work (Scheme 48A) [156], Fañanás and Rodríguez developed an intramolecular cationic cyclization strategy to access cyclohexenyl chlorides via chloride trapping of alkenyl cation intermediates (Scheme 48B) [157]. Comparable yields were obtained when either alkene or alcohol precursors were employed, consistent with the formation of a common cationic intermediate. The method proved limited to six-membered ring systems, with no successful extension to other ring sizes. The proposed mechanism involves initial generation of a tertiary or benzylic carbocation, followed by intramolecular cyclization with a pendant alkyne to furnish a vinyl cation intermediate. This species is postulated to be trapped by the solvent, potentially forming a transient chloronium ion (CH_2_Cl^+^). Reaction of this electrophilic intermediate with the BF_4_^−^ counterion would be expected to yield CH_2_FCl and BF_3_; however, the authors did not investigate or confirm the formation of these by-products.

In 2006, Cook reported a related cyclization initiated by the activation of allyl bromide using InCl_3_ (Scheme 48C) [158]. Very likely the first step involves the generation of allyl cation **I** by activation of the allyl bromide with InCl_3_. Subsequent trapping by the appended alkyne will form alkenyl cation **II**. Cation **II** then reacts with dichloromethane to yield the corresponding alkenyl chlorides. This observation suggests that, under the reaction conditions, InCl_3_Br^−^ exhibits lower nucleophilicity than dichloromethane. Increased dilution of the reaction with CH_2_Cl_2_ enhances the selectivity for chloride-to-bromide transfer, as evidenced by the ratio of Cl:Br shifting from 1:2 at 10 M CH_2_Cl_2_ to 29:1 at 0.1 M CH_2_Cl_2_. This finding indicates that non-indium coordinated solvent acts as the active nucleophile. The potential formation of chlorobromomethane in this reaction was not explored. When R = Ph (compound **248**), the formation of only alkenyl bromide **249** was observed. Several other instances of chloride abstraction from dichloromethane have been documented in the literature, particularly in reactions involving alkenyl cation intermediates [159–166].

**Scheme 49:**
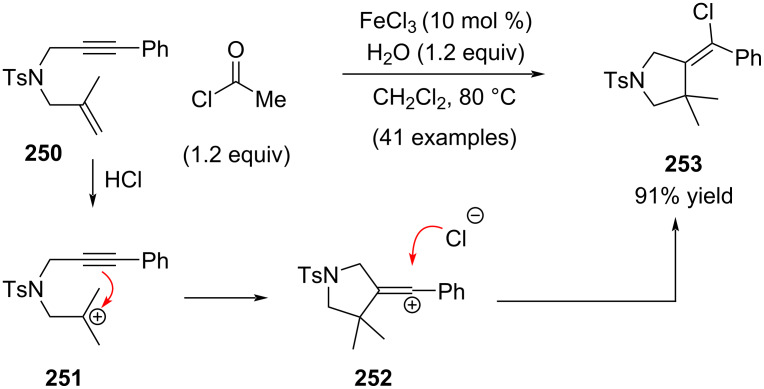
Intramolecular trapping reactions affording alkenyl chlorides.

In 2023, Li, Lou and You reported a closely related hydrochlorination–alkyne addition sequence with respect to Johnson, Fañanás, and Rodríguez works (Scheme 49) [167]. Acetyl chloride reacts with water to give HCl which then readily protonates trisubstituted alkene **250** [168–169]. Subsequent trapping of the tertiary aliphatic cation **251** by the adjacent alkyne furnishes the alkenyl cation intermediate **252**. Addition of chlorine, likely from FeCl_4_^−^ but potentially also from dichloromethane, to the alkenyl cation occurs from the less-hindered side, resulting in the formation of alkenyl chloride **253** in high yield.

**Scheme 50:**
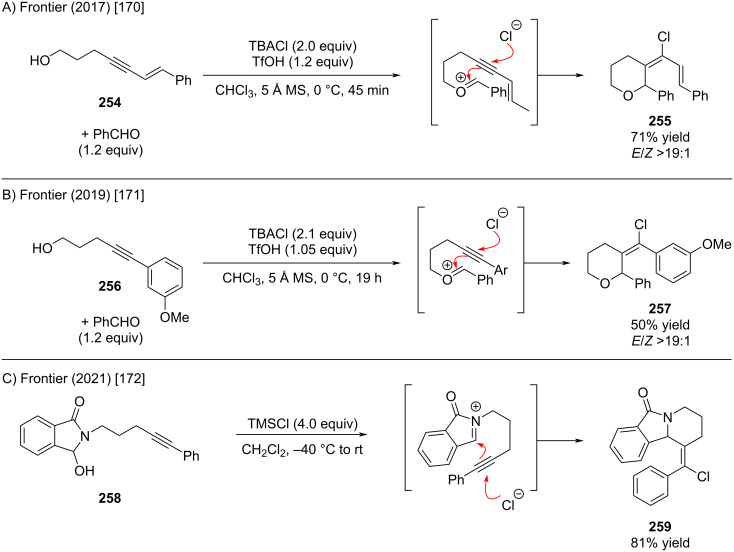
Intramolecular trapping reactions of oxonium and iminium ions affording alkenyl chlorides.

The Frontier group recently disclosed an alkynyl halo-(aza)-Prins cyclization strategy (Scheme 50) [170–172]. This transformation furnishes synthetically versatile cyclized products bearing pendant halide functionalities, which can serve as substrates for subsequent halo-Nazarov cyclizations. The sequential nature of this approach enables rapid construction of structurally complex motifs in a concise number of steps. A recent account by Frontier and Hernandez provides a comprehensive and mechanistically summary of halo-Nazarov electrocyclizations [173].

#### 1.7 Coupling reactions

**Scheme 51:**
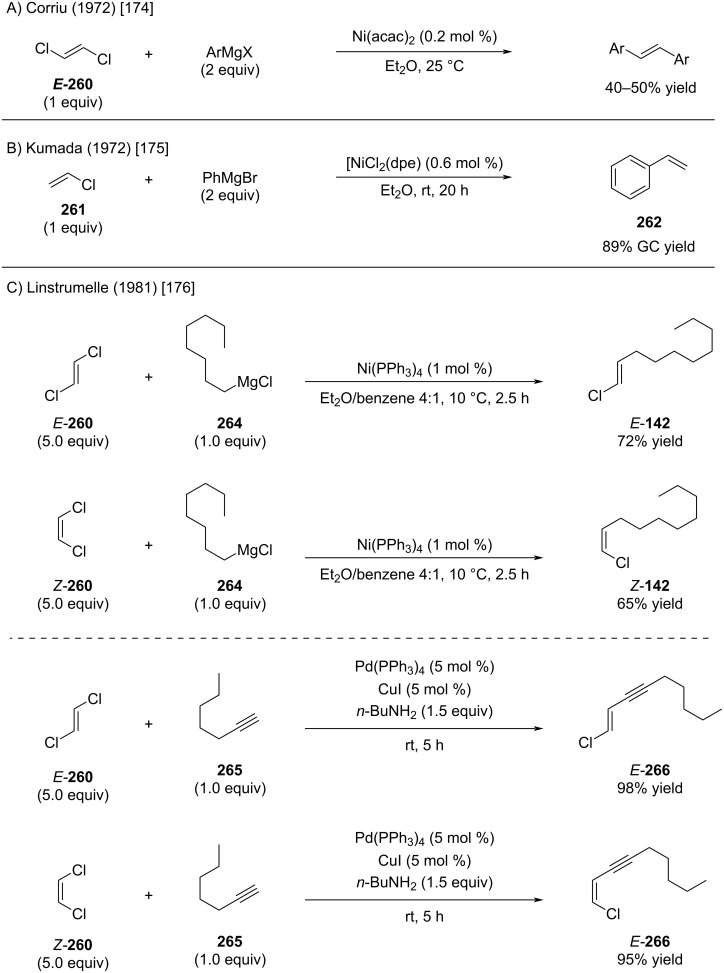
Palladium and nickel-catalyzed coupling reactions to afford alkenyl chlorides.

As already mentioned in the introduction, the coupling reaction of 1,1-dichloroalkenes was recently covered by Guinchard and Roulland [33]. Key publications in this area are highlighted as follows. To the best of our knowledge, Corriu was the first to utilize alkenyl chlorides in nickel-catalyzed coupling reactions with aryl Grignard reagents (Scheme 51A) [174]. Shortly thereafter, Kumada reported a more comprehensive study involving coupling reactions of vinyl chloride with aryl Grignard reagents (Scheme 51B) [175]. The first instances of monocoupling reactions of 1,2-dichloroethylenes were reported by Linstrumelle, who leveraged stoichiometric control (using 5 equivalents of 1,2-dichloroethylene) to achieve selective monocoupling reactions catalyzed by nickel with Grignard reagents (Scheme 51C) [176]. Building on Sonogashira's findings [177], Linstrumelle also documented the first monoselective coupling of 1,2-dichloroethylenes with alkynes using palladium catalysis (Scheme 51C). Organ and Negishi demonstrated that the use of one equivalent of (*E*)-1-iodo-2-chloroethylene enables selective Sonogashira reactions, yielding alkenyl chlorides in high yields (not shown) [178–180].

**Scheme 52:**
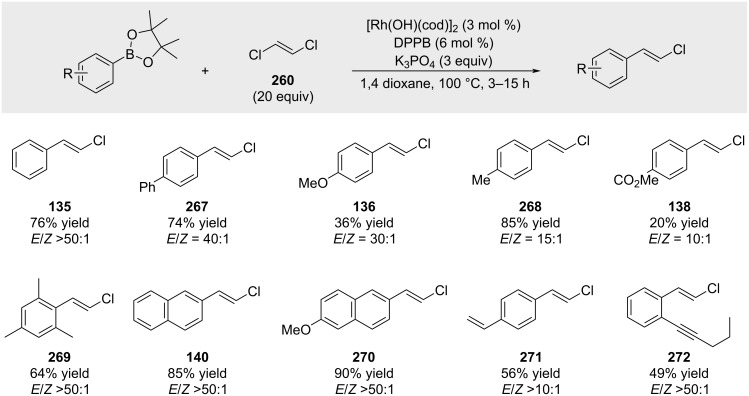
Rhodium-catalyzed couplings of 1,2-*trans*-dichloroethene with arylboronic esters.

Matsuda and co-workers reported a rhodium-catalyzed coupling reaction of 1,2-*trans*-dichloroethene with arylboronic esters (Scheme 52) [181]. They discovered that a rhodium/1,4-bis(diphenylphosphino)butane (DPPB) complex, in combination with a large excess of 1,2-*trans*-dichloroethene, facilitated monoselective cross-coupling reactions.

**Scheme 53:**
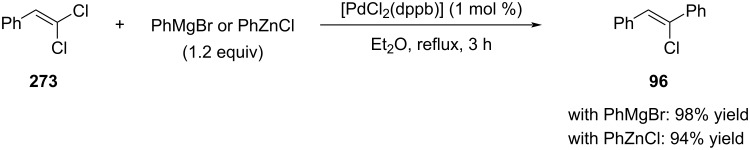
First report on monoselective coupling reactions for 1,1-dichloroalkenes.

In 1987, Minato, Suzuki, and Tamao reported the first monoselective coupling reactions for 1,1-dichloroalkenes (e.g., compound **273**, Scheme 53) [182].

**Scheme 54:**
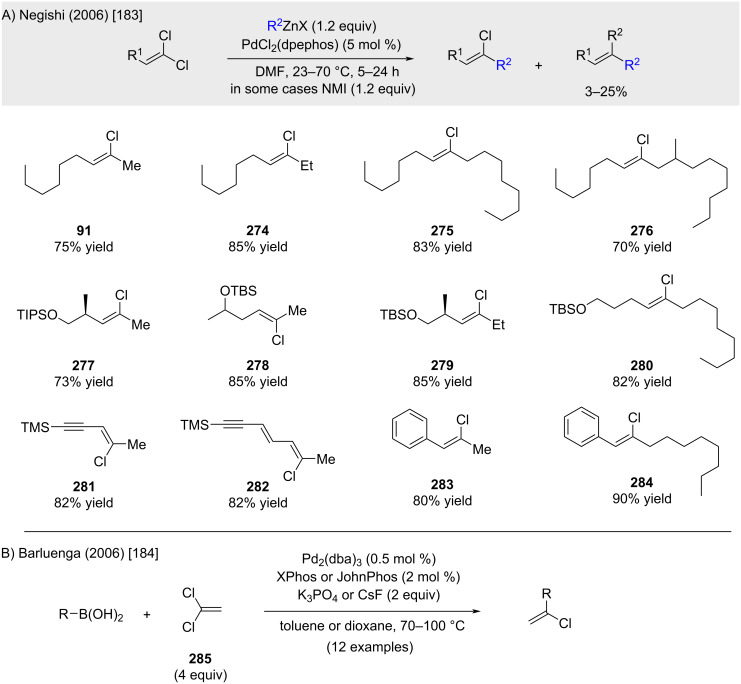
Negishi’s and Barluenga’s contributions.

However, as noted by Negishi, this procedure results in mixtures or exclusively dialkylation products when applied to alkyl-substituted 1,1-dichloro-1-alkenes. Negishi and co-worker reported an improved version of the procedure using one equivalent of *N*-methylimidazole (NMI) (Scheme 54A) [183]. However, the reaction still resulted in 3–25% yield of the dialkylated product. Consequently, the reaction conditions required adaptation for each substrate to maximize the yield of the monoalkylated product. In a parallel work, Barluenga demonstrated that the use of 4 equivalents of 1,1-dichloroethylene in combination with XPhos or JohnPhos ligands enabled monoselective cross-coupling with alkenyl- and arylboronic acids (Scheme 54B) [184].

Though mechanistically not related it is noteworthy that diazonium salts couple efficiently with alkenyl chlorides (not shown) [185–186].

#### 1.8 Alkenyl chloride synthesis via olefin metathesis

**Scheme 55:**
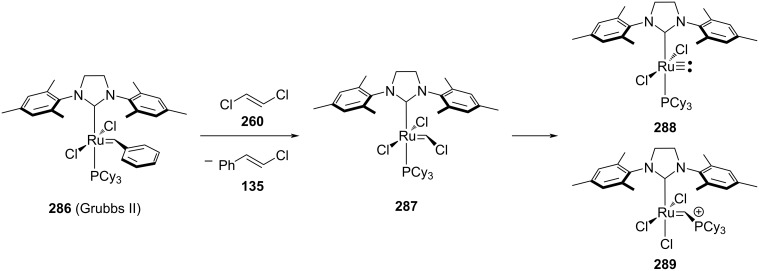
First mechanistic investigation by Johnson and co-workers.

Johnson and co-workers investigated complex **287**, which forms as a product of an initial metathesis cycle in the cross-metathesis (CM) with alkenyl chlorides (Scheme 55) [187–188]. The authors observed that complex **287** rapidly decomposes into catalytically inactive species **288** and **289** (Scheme 55). Consequently, metathesis with *trans*-chloroethylene presents a significant challenge.

**Scheme 56:**
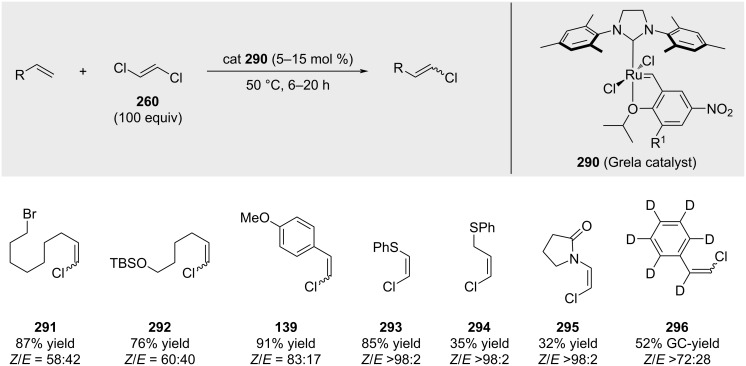
First successful cross-metathesis with choroalkene **260**.

Grela was the first to demonstrate that metathesis is indeed feasible using phosphine-free catalysts [189]. The formation of the desired alkenyl chloride product was favored by the utilization of a large excess (100 equivalents) of (*E*)-1,2-dichloroethylene in the presence of the Grela catalyst **290** [190]. Depending on the substrate, stereoselectivity and yields can vary significantly (Scheme 56).

**Scheme 57:**
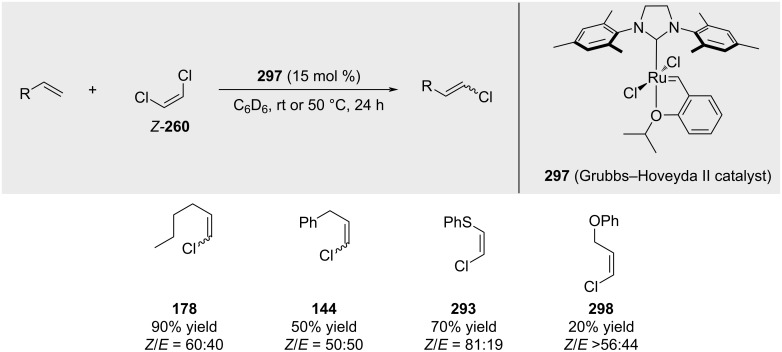
Subsequent studies by Johnson.

A subsequent study by Johnson confirmed Grela's findings using the standard Grubbs–Hoveyda 2nd generation catalyst **297** (Scheme 57) [188].

**Scheme 58:**
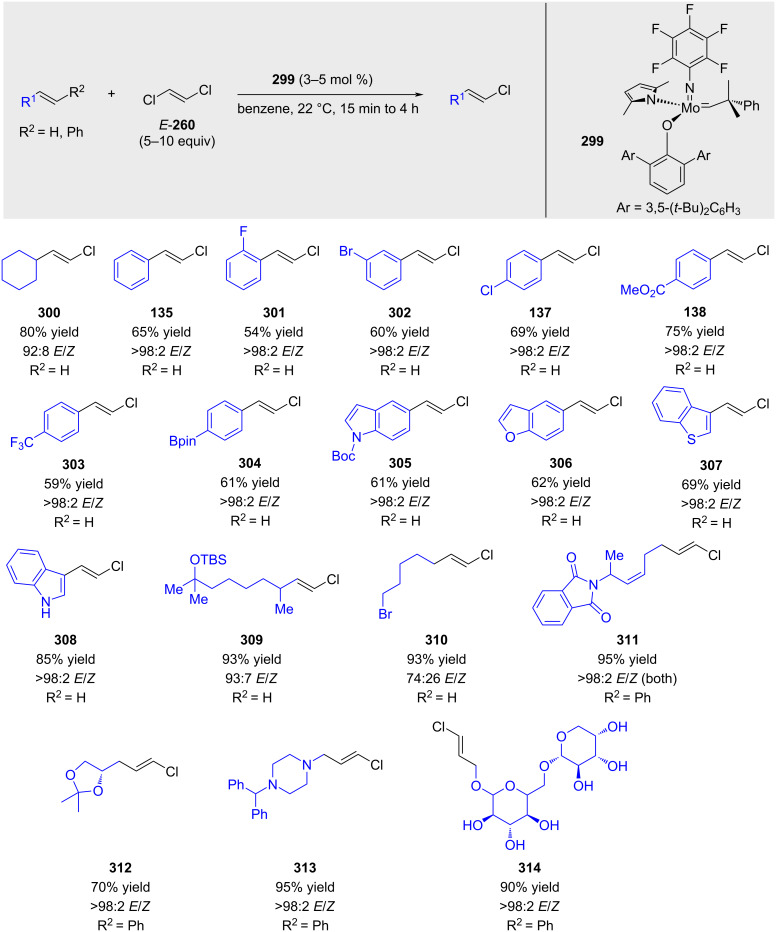
Hoveyda and Schrock’s work on stereoretentive cross-metathesis with molybdenum-based catalysts.

Schrock and Hoveyda reported a stereoretentive cross-metathesis reaction using commercially available *trans*-1,2-dichloroethene (Scheme 58) [191]. High yields and selectivities were achieved for alkenes with sterically demanding groups and styrenes. However, linear alkenes were obtained with low stereoretention.

**Scheme 59:**
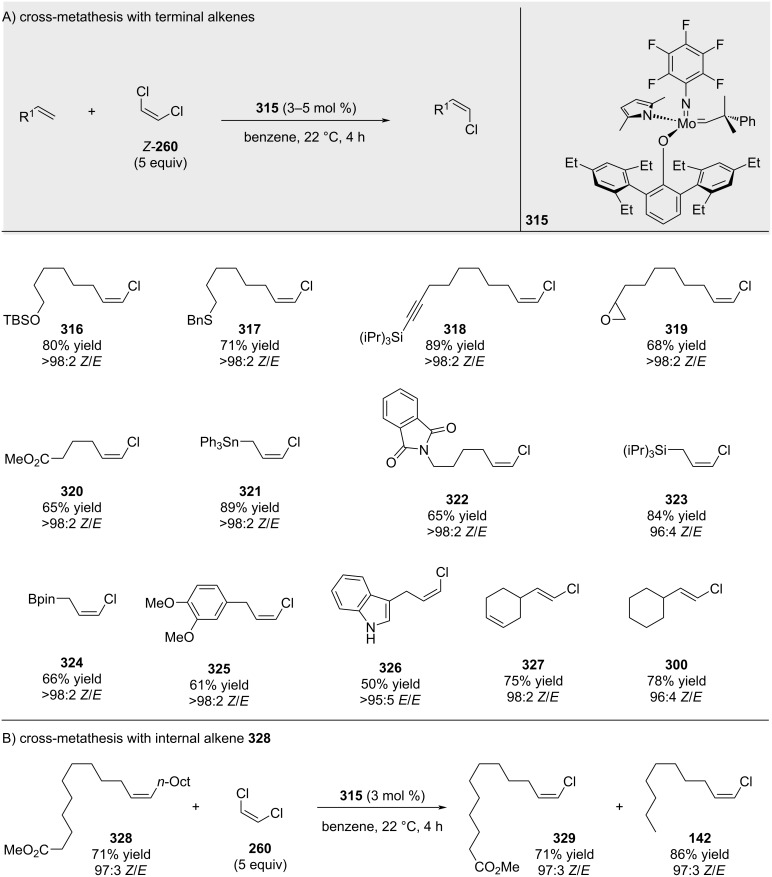
Related work with (*Z*)-dichloroethene.

In contrast to (*E*)-dichloroethene, (*Z*)-dichloroethene exhibited high levels of stereoretentivity for aliphatic alkenes (Scheme 59) [192]. Reactions with styrenes were inefficient, regardless of their electronic attributes.

**Scheme 60:**
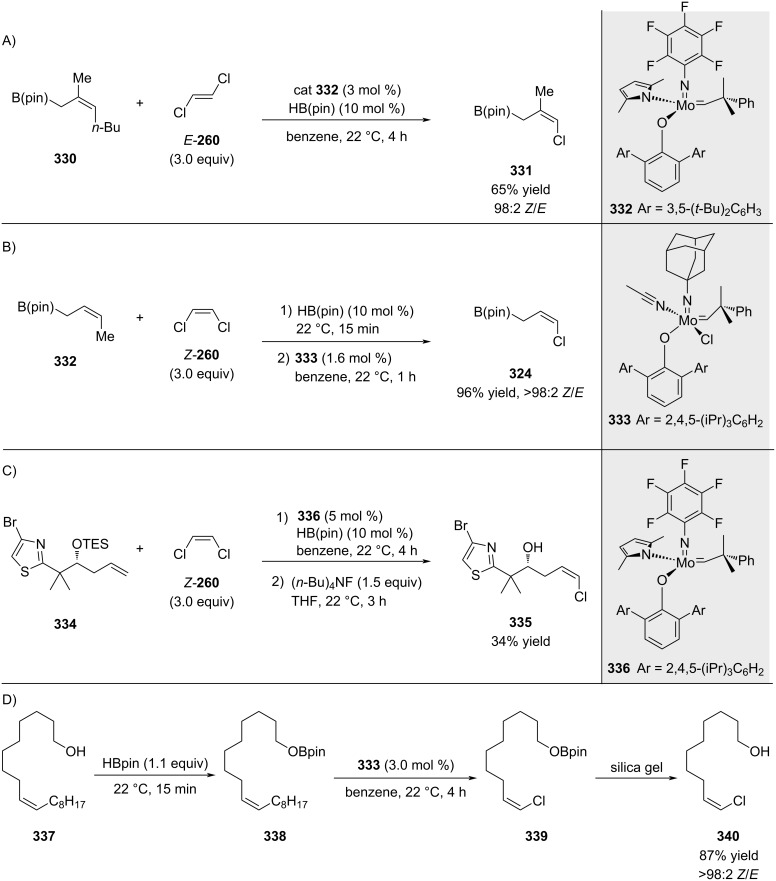
Further ligand refinement and traceless protection of functional groups with HBpin.

Further improvement of the reaction was achieved by fine-tuning the Mo-metathesis catalyst (Scheme 60A–C). Schrock and Hoveyda also demonstrated that adding small amounts of HB(pin) helps remove residual water, significantly enhancing the reaction efficiency [193]. This technique can also be applied to hydroxy- or carboxylic acid-containing olefins by adding 1.1 equivalents of HB(pin) to perform traceless protection of these functional groups, which are otherwise incompatible with molybdenum metathesis catalysts. The in situ-generated boronic ester is conveniently cleaved by silica gel (Scheme 60D).

As mentioned in the introduction, a comprehensive account of this work has been recently reported by Hoveyda [39].

#### 1.9 Olefinations of ketones and aldehydes

**Scheme 61:**
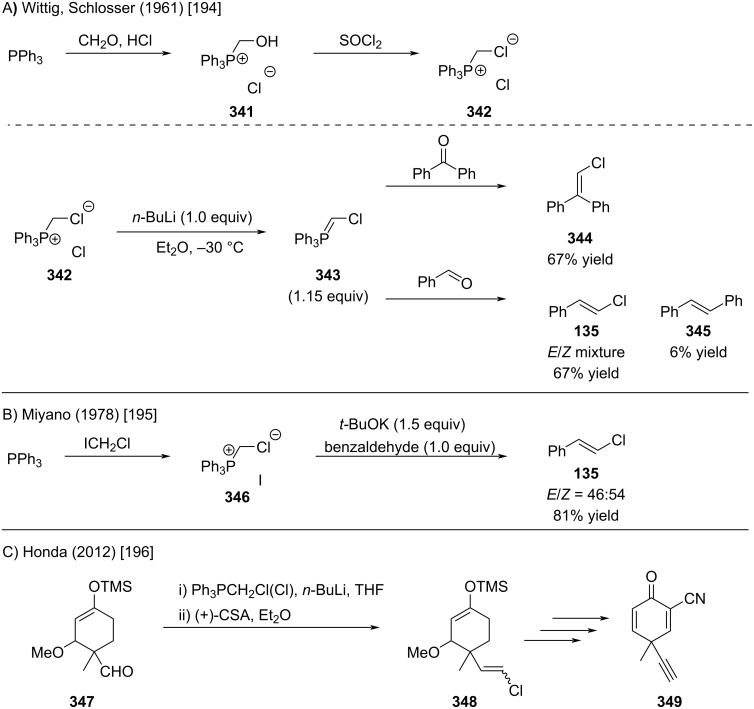
Alkenyl chloride synthesis by Wittig reaction.

In 1961, Wittig and Schlosser reported the first synthesis of alkenyl chlorides via the classical Wittig reaction (Scheme 61A) [194]. The Wittig reagent was prepared by reacting triphenylphosphine with formaldehyde in the presence of HCl, followed by treating the resulting alcohol **341** with thionyl chloride (Scheme 61A). The phosphonium salt was deprotonated with *n*-BuLi, and the corresponding phosphonium ylide was reacted with benzophenone or benzaldehyde to obtain the corresponding alkenyl chlorides **344** and **135**. In 1978, Miyano reported an improved procedure that prepares the phosphonium salt in one step from chloroiodomethane and typically the products are obtained as mixtures of stereoisomers (Scheme 61B) [195]. Today, the phosphonium salt **342** is commercially available (CAS: 5293-84-5) and is frequently employed for the formation of alkenyl chlorides (e.g., Scheme 60C) [196]. It is noteworthy that a highly *cis*-selective variant, necessitating a sophisticated phosphonium salt, was reported by Schlosser in 1993 [197].

**Scheme 62:**
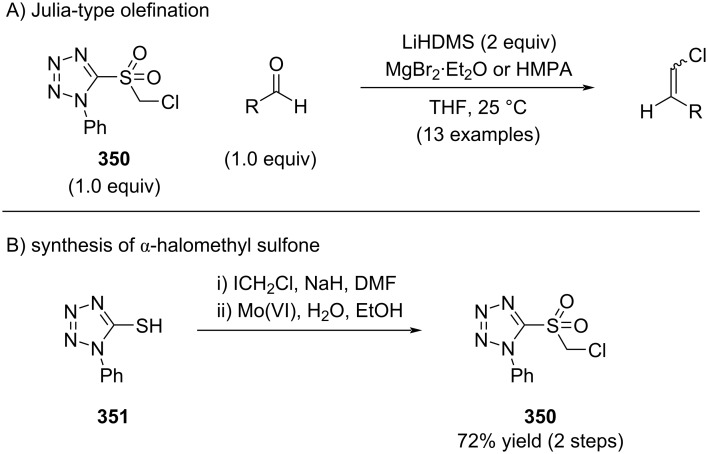
Alkenyl chloride synthesis by Julia olefination.

In 2006, Berthelette reported the Julia olefination of α-halomethylsulfones with aldehydes, which furnished alkenyl chlorides in good to excellent yields (Scheme 62A) [198]. The requisite α-halomethylsulfones were synthesized in two steps from commercially available reagents, achieving synthetically useful yields (Scheme 62B). Systematic optimization studies revealed that additives, such as magnesium bromide etherate or HMPA, had a pronounced impact on both reaction efficiency and stereoselectivity.

**Scheme 63:**
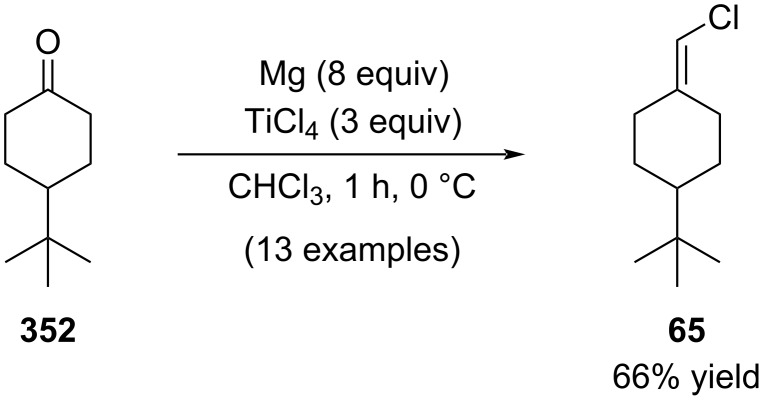
Alkenyl chloride synthesis by reaction of ketones with Mg/TiCl_4_ mixture.

In 2005, Yan reported that the direct oxidative addition of chloroform to a Mg–TiCl_4_ bimetallic system generates a nucleophilic chloromethylenetitanium species that transforms a ketone into the corresponding alkenyl chlorides (Scheme 63) [199].

#### 1.10 Allylic substitutions

**Scheme 64:**
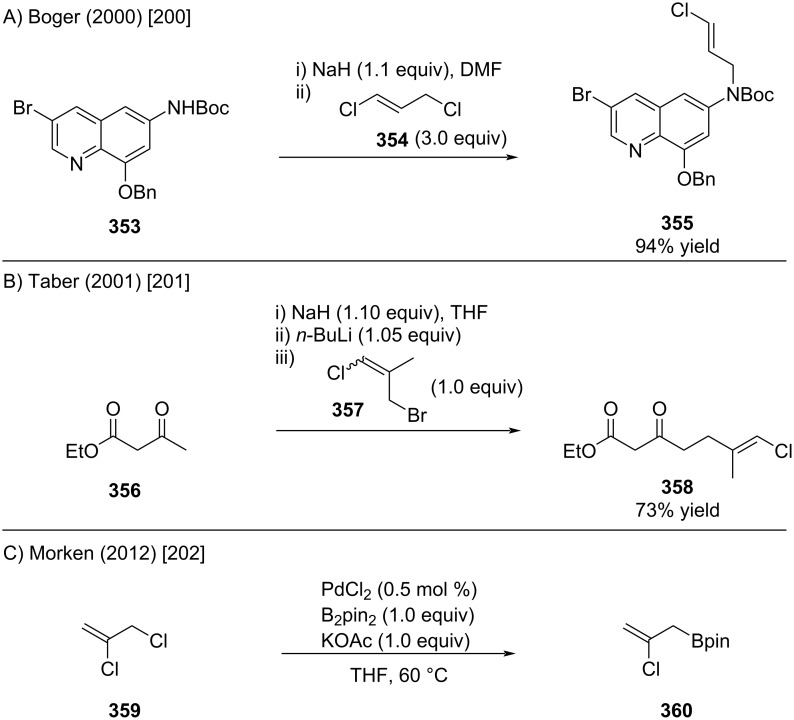
Frequently used allylic substitution reactions which lead to alkenyl chlorides.

Allylic substitution of readily accessible alkenyl chlorides bearing a suitable leaving group at the allylic position enables efficient introduction of the alkenyl chloride motif. Scheme 64, depicts representative examples by Boger [200], Taber [201], and Morken [202].

**Scheme 65:**
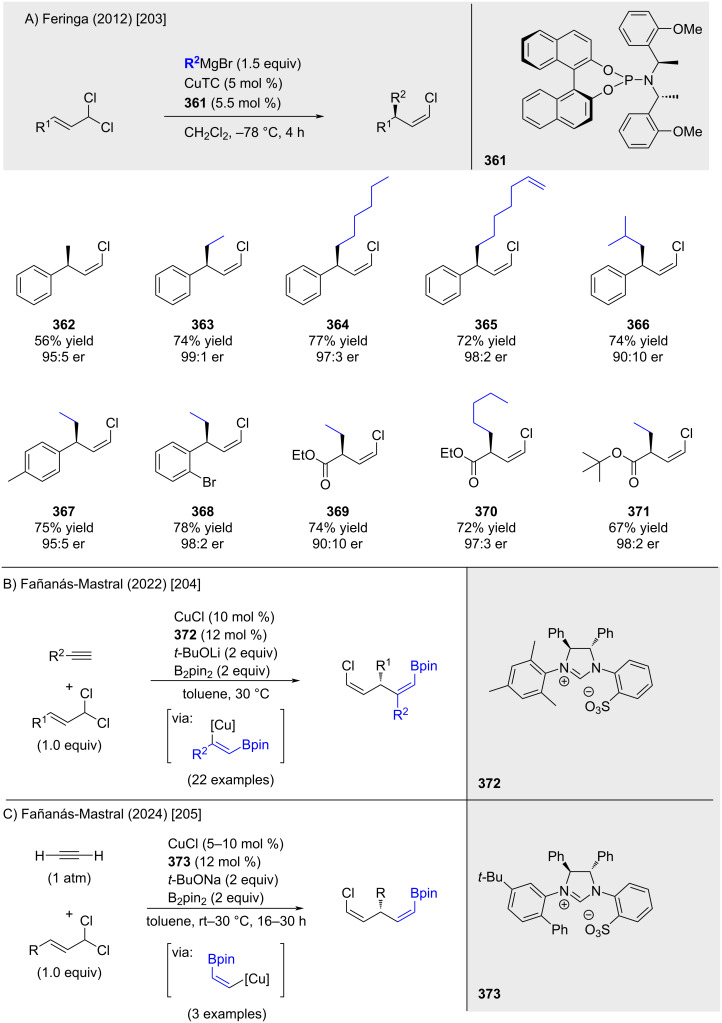
Enantioselective allylic substitutions.

In 2012, Feringa and co-workers reported the first enantioselective allylic substitution of allylic *gem*-dichlorides with alkyl Grignard reagents (Scheme 65A) [203]. The reaction, catalyzed by a chiral copper complex generated in situ from CuTC and phosphoramidite ligand **361**, furnished the corresponding enantioenriched alkenyl chlorides in high yield and with exclusive formation of the *Z*-isomer. A decade later, Fañanás-Mastral and co-workers demonstrated that alkenylcopper species, generated via borocupration of terminal alkynes, underwent allylic substitutions to furnish Bpin-substituted alkenyl chlorides in high enantioselectivity (Scheme 65B) [204]. Key to the transformation was the use of a sulfonate-substituted N-heterocyclic carbene ligand, introduced as its imidazolium salt precursor **372**. More recently, the same group extended this methodology to the use of acetylene as a highly useful C_2_-building block, further broadening the synthetic utility of this approach (Scheme 65C) [205].

It is worth noting that allylic substitution of allylic *gem*-dichlorides has recently been exploited in the preparation of peptidomimetics incorporating chloroalkene dipeptide isosteres [206].

#### 1.11 Miscellaneous reactions

**Scheme 66:**
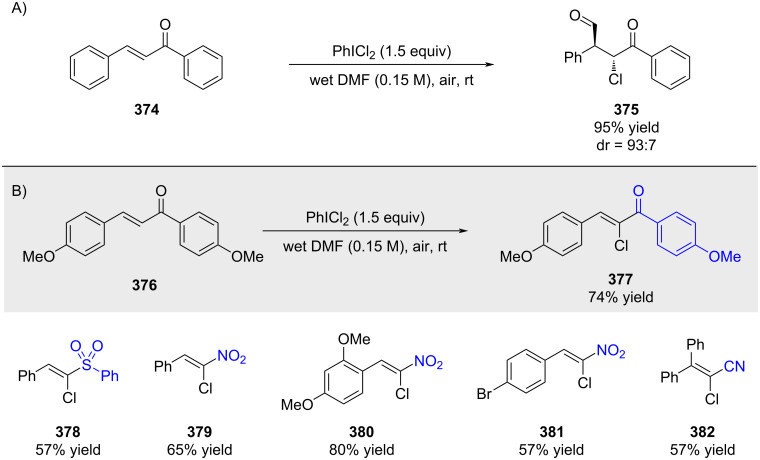
Synthesis of alkenyl chlorides bearing an electron-withdrawing group.

In 2014, Du and Zhao reported that PhICl_2_ in wet DMF was found transform alkenes regioselectively into the corresponding chloroformyloxylated products or α-chlorinated olefinic products, depending on the type of structure of the original unsaturated starting material (Scheme 66) [207].

**Scheme 67:**
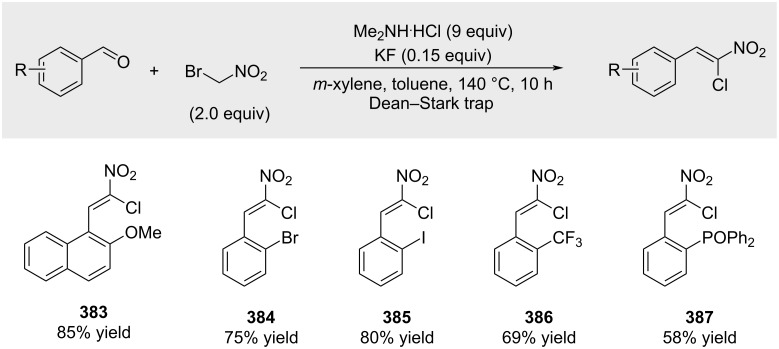
Synthesis of α-nitroalkenyl chlorides from aldehydes.

Similar products were obtained by Dauzonne and co-workers through the reaction of aldehydes with bromonitromethane in the presence of dimethylammonium chloride (not shown) [208]. Bonne and Rodriguez subsequently reported a slightly modified procedure in 2017 (Scheme 67) [209].

**Scheme 68:**
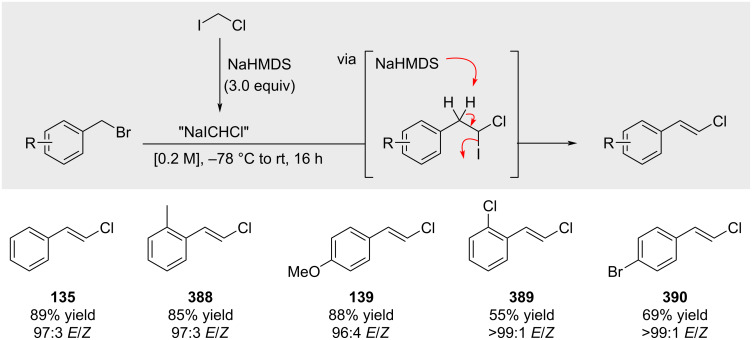
Synthesis of alkenyl chlorides via elimination of an in situ generated geminal dihalide.

In 2008, Charette reported that treatment of benzyl bromides with “NaICHCl” furnished the corresponding styrenyl chlorides in good yields and with high *E*-selectivity (Scheme 68) [210].

**Scheme 69:**
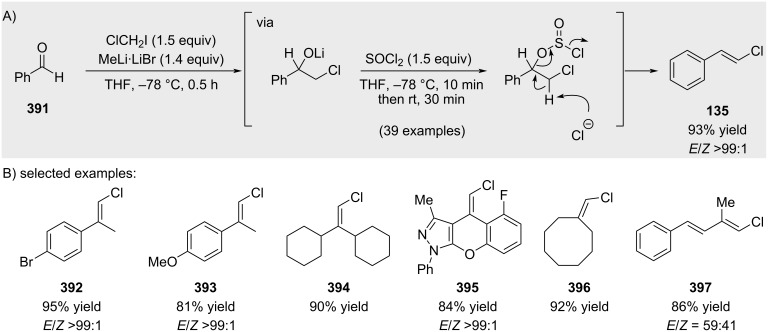
Carbenoid approach reported by Pace.

Pace and co-workers recently reported an alternative strategy for alkenyl chloride synthesis employing lithium chloromethylenecarbenoids generated in situ from chloroiodomethane and MeLi (Scheme 69) [211]. Nucleophilic addition of the carbenoid to ketones furnishes lithium alkoxides, which, upon treatment with SOCl_2_, undergo elimination to deliver alkenyl chlorides under mild basic conditions.

**Scheme 70:**
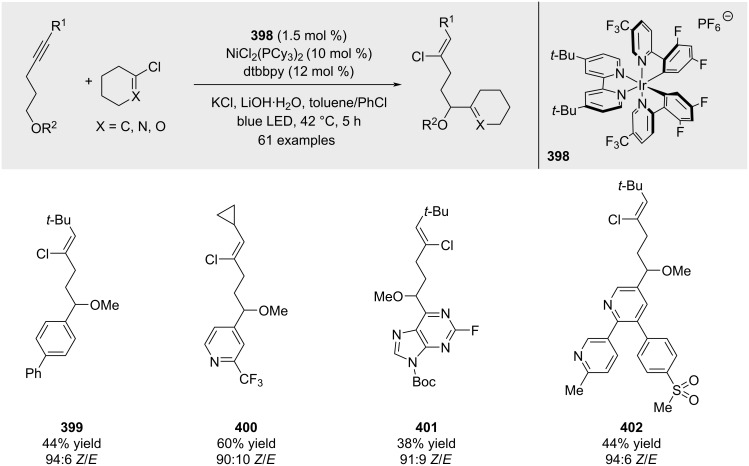
Carbenoid approach reported by Pace.

Chu and co-workers developed a dual photoredox/nickel catalytic system enabling the site- and stereoselective synthesis of γ-functionalized alkenyl chlorides from unactivated internal alkynes and various organochlorides (Scheme 70) [212].

**Scheme 71:**
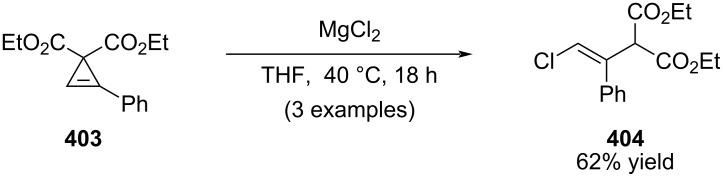
Ring opening of cyclopropenes in the presence of MgCl_2_.

In 2009, Lam and co-worker reported that bis-activated cyclopropenes undergo highly stereoselective ring-opening in the presence of stoichiometric magnesium halides to furnish multisubstituted alkenyl halides (Scheme 71) [213]. The transformation proceeds with excellent control of stereochemistry.

**Scheme 72:**
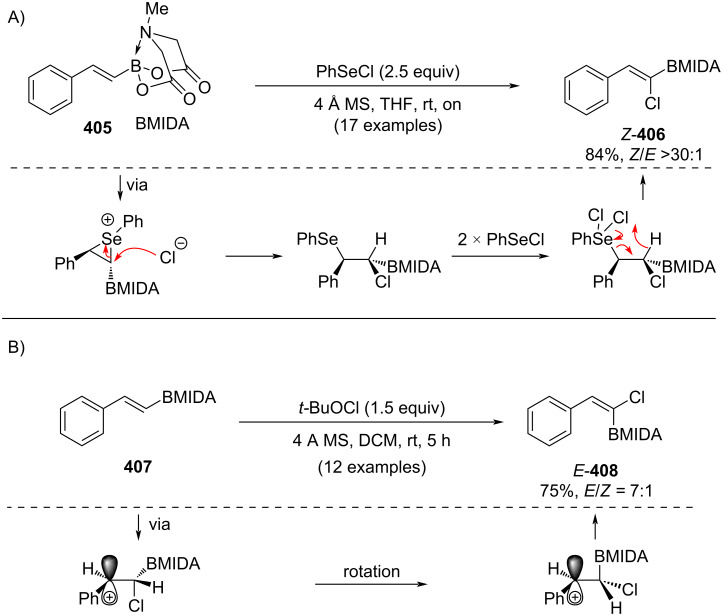
Electrophilic chlorination of alkenyl MIDA boronates to *Z*- or *E*-alkenyl chlorides.

In 2017, Wang and co-workers reported the electrophilic chlorination of alkenyl MIDA boronates as a stereoselective route to *Z*- or *E*-alkenyl chlorides [214]. Treatment with PhSeCl led predominantly to (*Z*)-chloroalkenes (Scheme 72A), while exposure to *t*-BuOCl furnished the corresponding *E*-isomers (Scheme 72B). Distinct mechanistic pathways were proposed for each transformation, supported by experimental studies.

**Scheme 73:**
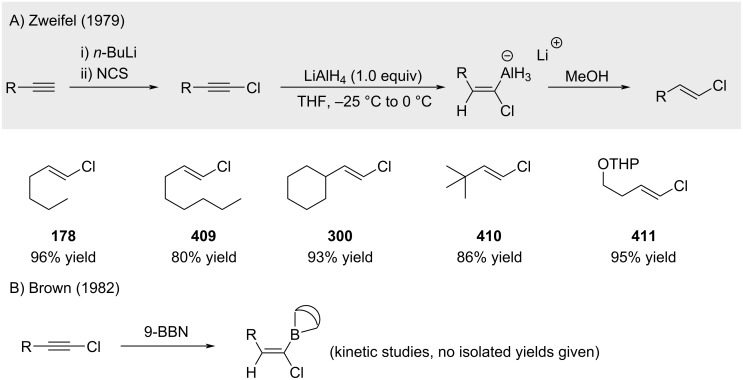
Hydroalumination and hydroboration of alkynyl chlorides.

Zweifel and Brown reported the synthesis of alkenyl chlorides via hydroalumination or hydroboration of chloroalkynes [215–216]. However, these methods are less practical relative to the direct reaction of alkenylmetal species with chlorine electrophiles (Scheme 73).

**Scheme 74:**
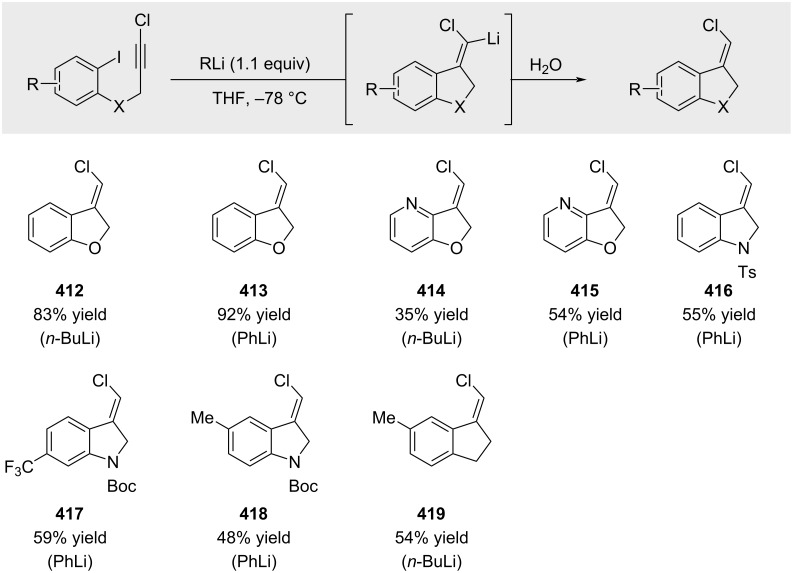
Carbolithiation of chloroalkynes.

Durandetti and Maddaluno reported that intramolecular carbolithiation of chloro-substituted alkynes provides access to exocyclic alkenyl chlorides upon aqueous work-up (Scheme 74) [217]. However, isolation is challenged by significant isomerization. Although crude yields are consistently high, exposure to silica gel induces isomerization of the exocyclic double bond to the corresponding internal isomer, resulting in substantially diminished isolated yields.

**Scheme 75:**
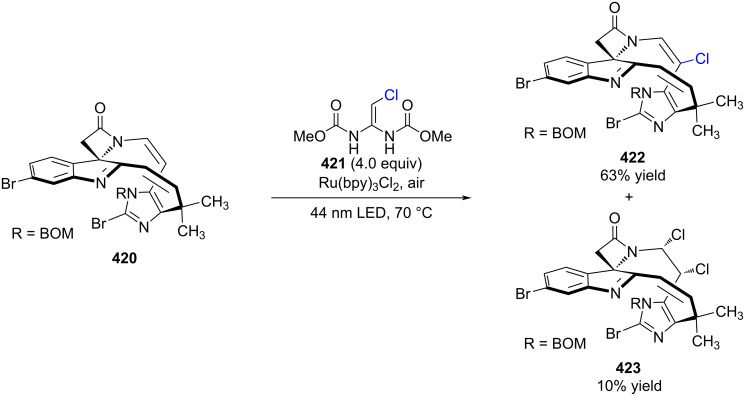
Chlorination of enamine **420**.

In the context of their total synthesis of chertelline C, Herzon and co-workers investigated a series of chlorination conditions (not shown). Among these efforts, a highly selective radical chlorination of an enamide was developed (Scheme 75), enabling site-specific chlorination under mild conditions [218].

### 2 Applications of alkenyl chlorides

#### 2.1 Eliminations

**Scheme 76:**
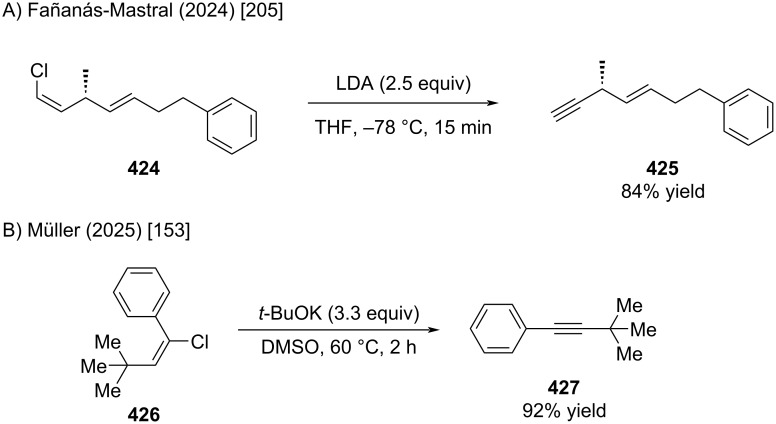
Alkyne synthesis by elimination of alkenyl chlorides.

Chloroalkenes, regularly serve for the introduction of terminal alkynes. Several reactions were already shown in this review (Schemes 1, 5, 10, and 16). A recent example by Fañanás-Mastral and Müller is shown in Scheme 76 [153,205].

#### 2.2 Reductive metalations

**Scheme 77:**
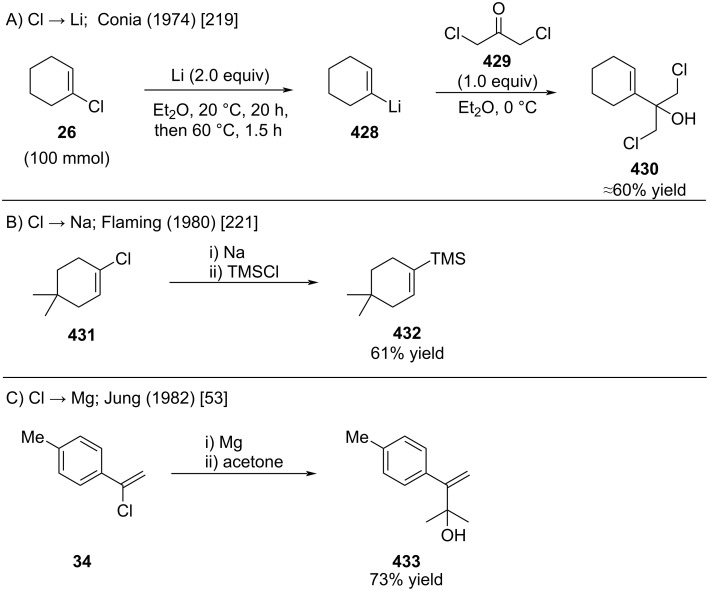
Reductive lithiation of akenyl chlorides.

Few protocols were reported for the transformation of alkenyl chlorides into the corresponding lithium, sodium, and magnesium reagents (Scheme 77). Conia reported the lithiation of alkenyl chloride **26** (Scheme 77A) [219]. An organic synthesis procedure for the synthesis of (*E*)-1-propenyllithium from (*E*)-1-chloropropene was reported by Linstrumelle and Whiteside in 1976 (not shown) [220]. Flaming reported the synthesis of an alkenyl sodium reagent from alkenyl chloride **431** (Scheme 77B) [221]. A detailed procedure of this transformation was subsequently reported by Nagendrappa [59]. Transformation of the sensitive alkenyl chloride (see Scheme 6C and discussion) into the corresponding Grignard reagent worked surprisingly well as demonstrated by Jung and co-workers (Scheme 77C) [53].

#### 2.3 Reaction of alkenyl chlorides with organolithium reagents

**Scheme 78:**
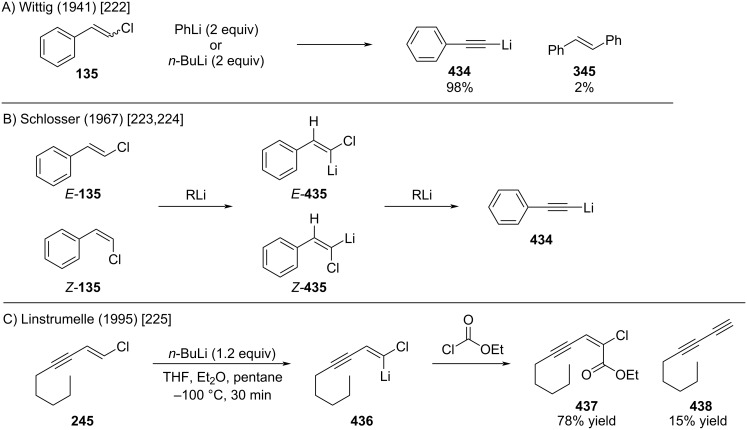
Reactions of alkenyl chlorides with organolithium reagents.

In 1941, Wittig and Witt reported that β-chlorostyrene (**135**) undergoes rapid elimination upon treatment with phenyl- or *n*-butyllithium, affording the corresponding lithium phenylacetylide **434** (Scheme 78A) [222]. In 1967, Schlosser conducted a detailed mechanistic study of this transformation (Scheme 78B). Kinetic analysis and isotope effect experiments with deuterated substrates revealed a stepwise pathway: initial deprotonation occurs at the α-position relative to the chlorine, followed by deprotonation at the β-position by a second equivalent of organolithium reagent to furnish lithium acetylide **434** [223–224]. In 1995, Linstrumelle reported a related work on chloroenynes (Scheme 78C) [225], which can be deprotonated at −100 °C with *n*-BuLi to generate the corresponding alkenyllithium reagents. These reagents react with various electrophiles, such as ethyl chloroformate, in good yields. Notably, metalation reactions with non-conjugated chloroalkenes or the corresponding (*Z*)-chloroenynes proved inefficient.

**Scheme 79:**
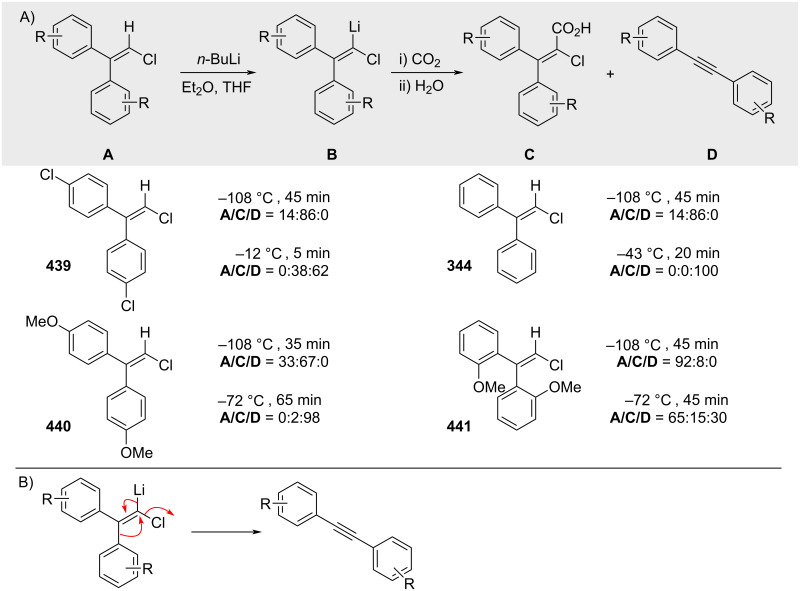
Reactions of alkenyl chlorides with organolithium reagents.

In 1964, Köbrich reported that treatment of alkenyl chlorides with *n*-butyllithium results in selective deprotonation at the position α to the chlorine (Scheme 79A) [226]. At low temperatures, the resulting intermediate can be intercepted by electrophiles such as CO_2_, affording the corresponding carboxylic acids upon aqueous work-up. At elevated temperatures, the major product observed is the corresponding alkyne. The formation of this product is attributed to elimination of chloride, proposed to proceed via aryl-assisted 1,2-migration and concurrent expulsion of lithium chloride (Scheme 79B).

**Scheme 80:**
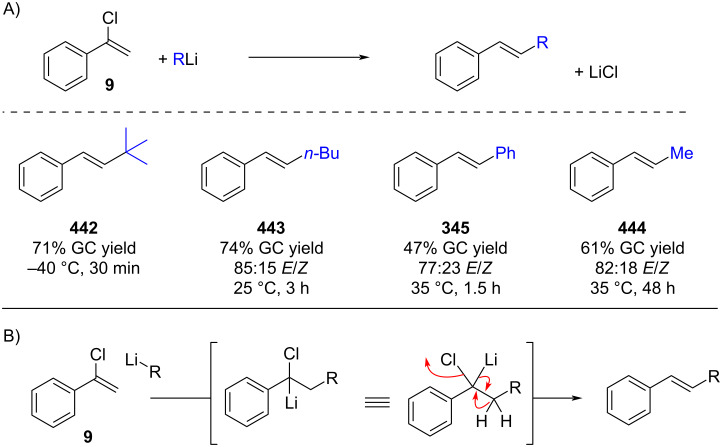
Addition–elimination reaction of alkenyl chloride **9** with organolithium reagents.

In 1975, Cunico reported the reaction of alkenyl chlorides with alkyl- and aryllithium reagents (Scheme 80A) [227]. Treatment of α-chlorostyrene (**9**) resulted in addition–elimination products. The authors proposed that the nucleophilic addition of the organolithium reagent to the alkenyl chloride generates a lithiumcarbenoid intermediate. This species then undergoes a 1,2-hydride shift followed by elimination of lithium chloride, analogous to the mechanism proposed by Köbrich (Scheme 80B). Collectively, these studies underscore the reluctance of alkenyl chlorides – unlike their bromide counterparts – to undergo halogen–lithium exchange. Instead, they preferentially engage in pathways involving either deprotonation or addition reactions.

**Scheme 81:**
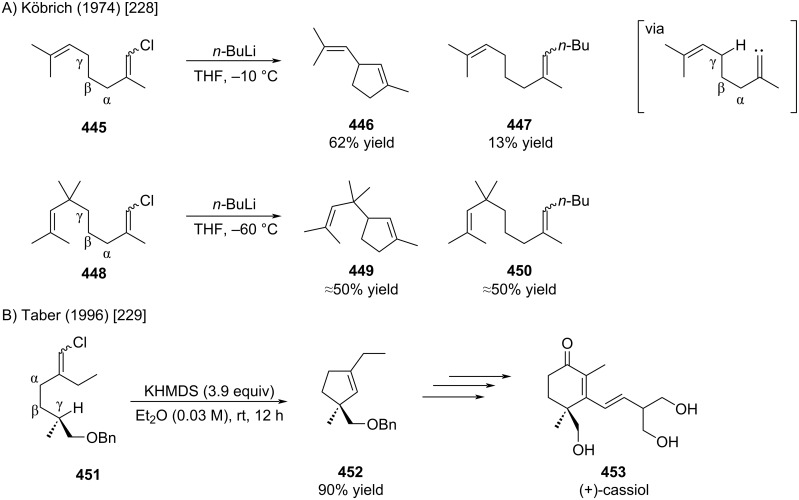
C–H insertions of lithiumcarbenoids.

In 1974, Köbrich showed that chloroalkenes possesing a γ-hydrogen can undergo cyclization by C–H insertion of the corresponding carbenoid (Scheme 81A) [228]. This reaction was subsequently utilized in the context of several total syntheses, for example as illustrated by Taber for the synthesis of (+)-cassiol (e.g., Scheme 81B) [229]. The chemistry of these carbenoid C–H insertions was reviewed by Marek, Knorr, and Grainger [230–232].

#### 2.4 Coupling reactions

**Scheme 82:**
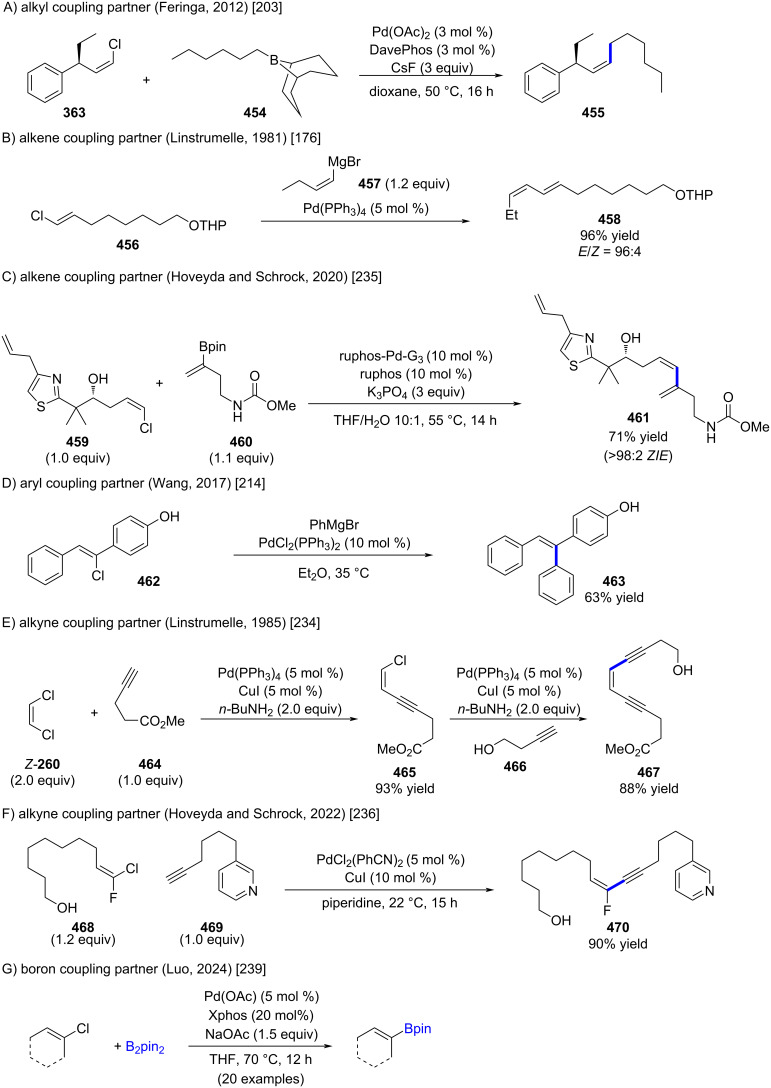
Pd-catalyzed coupling reactions with alkenyl chlorides as coupling partner.

**Pd-catalyzed coupling reactions:** As transition-metal-catalyzed coupling reactions have been discussed in chapter 1.7, this section highlights only selected complementary examples. While alkenyl chlorides are generally less reactive than their iodide or bromide counterparts [233], numerous successful palladium-catalyzed couplings with alkyl, alkenyl, aryl, and alkynyl nucleophiles have been reported. Representative cases are shown in Scheme 82 [176,203,214,234–236]. Sonogashira couplings of alkenyl chlorides are generally limited to (*E*)- or (*Z*)-1,2-dichloroethylenes and conjugated systems, where Pd(PPh_3_)_4_ gives good results (see Scheme 51C). However, in most other cases, alkenyl chlorides are significantly less reactive than their bromide or iodide analogues. In 1991, Linstrumelle and co-workers addressed this limitation by introducing a PdCl_2_(PhCN)_2_/CuI system in piperidine, enabling efficient coupling [237–238]. This catalytic system was recently applied by Hoveyda and Schrock (Scheme 82F) [236].

In 2024, Luo and co-workers reported a palladium-catalyzed synthesis of cyclic alkenylboronates from alkenyl chlorides (Scheme 82G) [239].

**Scheme 83:**
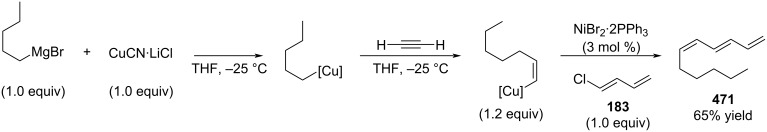
Ni-catalyzed coupling of alkenylcopper reagent with alkenyl chloride **183**.

**Ni-catalyzed coupling reactions:** Alexakis, Normant, and Fugier reported the synthesis of compound **471 –** the principal odoriferous component of Galbanum essential oil – via a nickel-catalyzed coupling between alkenyl chloride **183** and the corresponding alkenylcopper reagent (Scheme 83) [130]. Additional Ni-catalyzed coupling reactions with metalated arenes were outlined in Scheme 50.

**Scheme 84:**
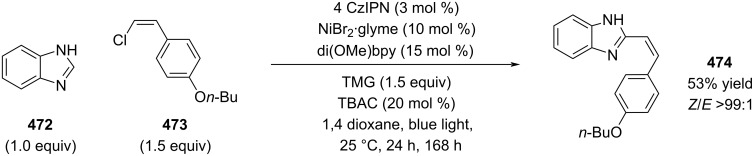
Ni-catalyzed coupling of heterocycle **472** with alkenyl chloride **473**.

Kurahashi recently reported a nickel–photoredox-catalyzed stereoconvergent coupling of alkenyl chlorides with nitrogen heterocycles, including one isolated example with alkenyl chloride **473** (Scheme 84) [240]. Notably, this substrate required a seven-fold longer reaction time compared to the corresponding bromides and iodides.

**Electrochemical coupling reactions:** It should be noted, that the use of alkenyl chlorides in electrochemical coupling reactions was recently reviewed by Marjani and co-workers (not shown) [241].

#### 2.5 Miscellaneous applications

**Scheme 85:**
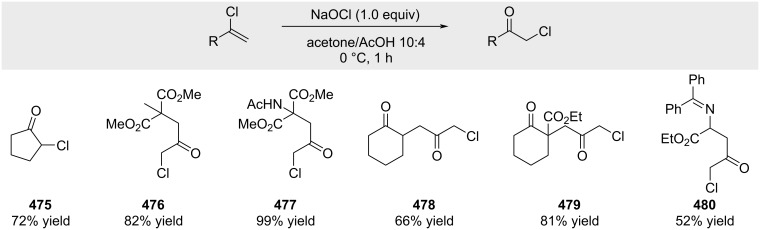
Synthesis of α-chloroketones by oxidation of alkenyl chlorides.

Weinreb and co-workers demonstrated that α-chloroketones can be synthesized from alkenyl chlorides by reaction with aqueous sodium hypochlorite in AcOH/acetone (Scheme 85) [242].

**Scheme 86:**
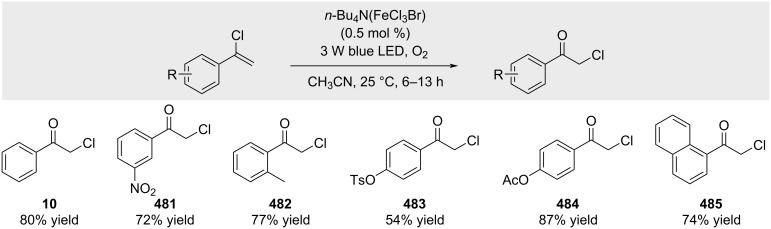
Tetrahalogenoferrate(III)-promoted oxidation of alkenyl chlorides.

Jiang and co-workers introduced tetrahalogenoferrate(III) complexes as a new class of iron photocatalysts (Scheme 86) [243]. Prepared from FeCl_3_ and simple bromide salts, these complexes show tunable photosensitivity in the visible range. Their distinct reactivity compared to FeCl_3_ enables aerobic oxidative transposition of alkenyl chlorides, providing α-chloroketones in high efficiency under mild conditions and with broad scope.

**Scheme 87:**
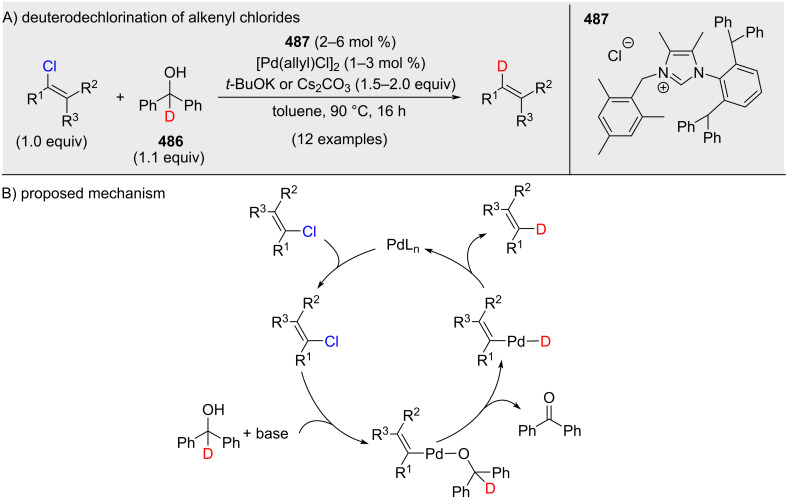
Chlorine–deuterium exchange promoted by a palladium catalyst.

Kuriyama and Onomura developed a Pd-catalyzed deuterodechlorination of alkenyl chlorides (Scheme 87) [56]. The method enables precise incorporation of deuterium, tolerates heterocycles and drug-derived scaffolds, and was demonstrated on gram scale for a deuterated iminostilbene core.

**Scheme 88:**
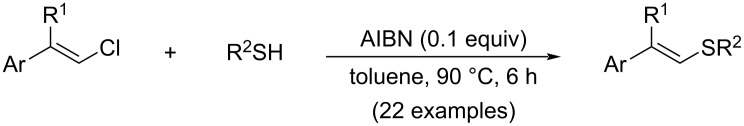
Reaction of alkenyl chlorides with thiols in the presence of AIBN (azobisisobutyronitrile).

In 2019, Shi and Cao reported a radical-promoted reaction of alkenyl chlorides with aliphatic and aromatic thiols to provide alkenyl sulfides (Scheme 88) [244].

**Scheme 89:**
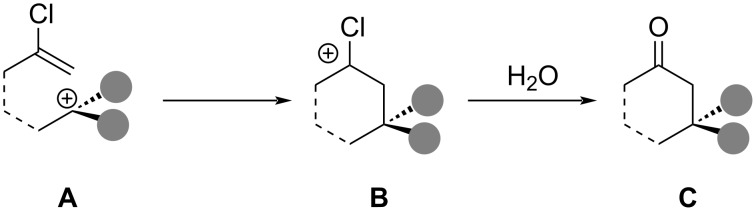
Chloroalkene annulation.

Chloroalkene annulations (Scheme 89) were reviewed by Lansbury several decades ago [245]. These transformations involve the intramolecular reaction of chloroalkene nucleophiles with in situ-generated tertiary or benzylic carbocations. The resulting cationic intermediate **B** typically provides the corresponding ketone after aqueous work‐up.

## Conclusion

This review represents my attempt to classify and structure the rapidly growing field of alkenyl chloride chemistry. I do not claim full coverage, yet I believe that the key reactions have been included to guide future work and help authors identify prior contributions. My survey also revealed that many recent publications overlooked related studies, and the true extent of this field remains underappreciated. While the synthesis of alkenyl chlorides is versatile, their synthetic applications – beyond Pd-catalyzed couplings – are still underdeveloped, offering ample opportunities for future synthetic discoveries.

## Data Availability

Data sharing is not applicable as no new data was generated or analyzed in this study.
